# The Role of P2X7 Receptor in Alzheimer’s Disease

**DOI:** 10.3389/fnmol.2020.00094

**Published:** 2020-06-03

**Authors:** Linda Francistiová, Carolina Bianchi, Caterina Di Lauro, Álvaro Sebastián-Serrano, Laura de Diego-García, Julianna Kobolák, András Dinnyés, Miguel Díaz-Hernández

**Affiliations:** ^1^BioTalentum Ltd., Gödöllõ, Hungary; ^2^Szent István University, Gödöllõ, Hungary; ^3^Department of Biochemistry and Molecular Biology, Veterinary School, Complutense University of Madrid, Madrid, Spain; ^4^Instituto de Investigación Sanitaria del Hospital Clínico San Carlos, Madrid, Spain; ^5^HCEMM-USZ StemCell Research Group, University of Szeged, Szeged, Hungary

**Keywords:** amyloidogenic processing, inflammation, oxidative stress, synaptopathy, microglia, induced pluripotent stem cells

## Abstract

Alzheimer’s disease (AD) is the most prevalent neurodegenerative disease characterized by a progressive cognitive decline associated with global brain damage. Initially, intracellular paired helical filaments composed by hyperphosphorylated tau and extracellular deposits of amyloid-β (Aβ) were postulated as the causing factors of the synaptic dysfunction, neuroinflammation, oxidative stress, and neuronal death, detected in AD patients. Therefore, the vast majority of clinical trials were focused on targeting Aβ and tau directly, but no effective treatment has been reported so far. Consequently, only palliative treatments are currently available for AD patients. Over recent years, several studies have suggested the involvement of the purinergic receptor P2X7 (P2X7R), a plasma membrane ionotropic ATP-gated receptor, in the AD brain pathology. In this line, altered expression levels and function of P2X7R were found both in AD patients and AD mouse models. Consequently, genetic depletion or pharmacological inhibition of P2X7R ameliorated the hallmarks and symptoms of different AD mouse models. In this review, we provide an overview of the current knowledge about the role of the P2X7R in AD.

## Highlights

- Alzheimer’s disease (AD) is a multifactorial neurodegenerative disorder.
- P2X7R is upregulated in AD.
- P2X7R is involved in microglial function, synaptopathy, oxidative stress, and amyloidogenic APP processing.
- Induced pluripotent stem cell (iPSC) is a promising new therapeutic approach in AD.

## Alzheimer’s Disease

Alzheimer’s disease (AD) is a devastating neurodegenerative disorder currently affecting more than 47 million people around the world, expecting to reach more than 131 million by 2050. Typical AD onset is after 65 years old, although in less than 5% of cases, onset may be earlier (Alzheimer’s-Association, 2019). Approximately between 1 and 3% of AD patients present autosomal dominant form of AD, denominated early familiar AD (eFAD) (Price and Sisodia, 1998). This form is characterized by mutations in both amyloid-β (Aβ) precursor protein (APP) and enzymes related in its processing, like presenilin-1 and presenilin-2 (PSEN1 and PSEN2) (Price and Sisodia, 1998; Ling et al., 2003).

Symptoms associated to AD follow a progressive course, starting with an impairment in learning and memory, proceeding to later detriments in complex attention, executive functions, language, visuospatial compartment, praxis, gnosis, behavior, and/or social compartment (McKhann et al., 2011). At neuropathological level, postmortem brains from AD patients show atrophy of frontotemporal cortex and hippocampus caused by neuronal loss, neuroinflammation, loss of synapses, and oxidative stress. Typical hallmarks of AD are extracellular senile plaques and intracellular neurofibrillary tangles (NFTs) (Gahtan and Overmier, 1999; Selkoe, 2001; Avila, 2006; Perl, 2010). Senile plaques are formed by β-amyloid Aβ peptides, generated by the sequential proteolysis of APP by β-secretase 1 (BACE1) and γ-secretase (PSEN1 and 2, Nicastrin, and APH-1) (Selkoe, 2001). NFTs are assembled by abnormal accumulation of hyperphosphorylated tau protein [microtubule associated protein tau (MAPT)] (Avila, 2006). Aβ peptide and phosphorylated Tau protein, primary criteria for AD diagnosis, are considered the main toxic species involved in AD (Long and Holtzman, 2019). Indeed, detection of Aβ and tau deposition in cerebrospinal fluid (CSF) or positron emission tomography (PET) imaging presents now an antemortem AD neuropathology diagnosis (Bridel et al., 2019; Lowe et al., 2019).

### Current Therapeutic Strategies in AD

There are only four commercial palliative-treatments available for symptomatic AD patients: three acetylcholinesterase inhibitors (donepezil, rivastigmine, galantamine) and memantine, a non-competitive NMDA receptors modulator (Long and Holtzman, 2019). Despite all efforts made, there is no effective treatment available for symptomatic AD patients. Over the last decade, numerous clinical trials have been carried out to avoid the amyloid toxicity associated with AD. One of those was focused on developing specific monoclonal antibodies against Aβ, both soluble and fibrillar forms (Doody et al., 2013; Salloway et al., 2014; Selkoe, 2019) or try to induce an active immunization. Other strategies have attempted to reduce brain Aβ burden designing new potent secretase inhibitors, both against γ-secretase or BACE1 inhibitors (Egan et al., 2018, 2019; Henley et al., 2019; Lopez Lopez et al., 2019). Another trial used anti-inflammatory drugs to avoid Aβ-induced neuroinflammation, focusing on the inhibition of the cyclooxygenase enzyme (Aisen et al., 2000; de Jong et al., 2008). Since some studies have shown increased levels of cholesterol promoting the production of Aβ, other clinical trials used statins likes hydroxymethylglutaryl coenzyme A (HMG-CoA) reductase inhibitors as cholesterol-lowering agents (Carlsson et al., 2008; Feldman et al., 2010). Regarding Tau-based clinical trials, the followed strategies have been focused on reducing its intracellular phosphorylation rate (Domínguez et al., 2012), avoiding its aggregation (Wischik et al., 1996, 2015), or allowing its removal using immunotherapy approaches. Tau immunotherapy is based on both active and passive immunization approaches. Active immunization must be handled carefully, avoiding the pathogenic activation of the immune system, in particular T-cells, that could lead to aseptic meningo-/encephalitis endangering patient safety. In contrast, passive immunotherapy provides the advantage of control over antibodies’ binding properties and their blood concentrations (Vogels et al., 2019). So far, nine ongoing immunotherapy studies are being reported (Alzforum, 2019), two of which are active immunotherapies: AADvac1 (Novak et al., 2017) and ACI-35 (Theunis et al., 2013); and seven passive immunotherapies: R07105705 (Lee et al., 2016), Zagotenemab (LY3303560) (Alam et al., 2017), BIIB076 (Czerkowicz, 2017), ABBV-8E12 (C2N 8E12) (Yanamandra et al., 2015), Gosuranemab (BIIB092) (Boxer et al., 2019), UCB0107 (Alzforum, 2018), and JNJ-63733657 (Alzforum, 2018). While there are several clinical trials ongoing (Long and Holtzman, 2019), complementary approaches targeting alternative pathways still need to be explored.

**FIGURE 1:**
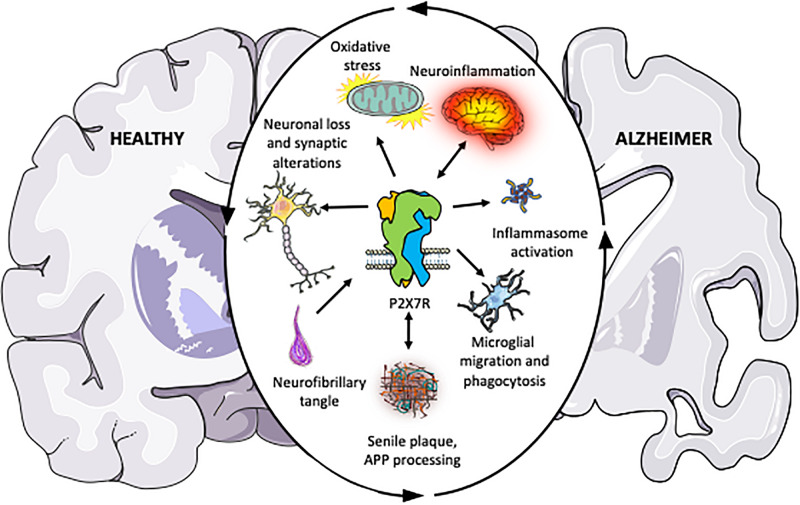
Schematic illustration summarizing the pieces of evidence accumulated over the past years indicating that P2X7R plays a central role in the different physiopathological processes associated with Alzheimer’s disease. The outer circular arrows illustrate a chain of interconnected and mutually influenced pathological processes associated with AD. Inner arrows represent the relationships found between P2X7R and these pathological processes, summarizing the studies discussed in the present review. Briefly, P2X7R modulates amyloid APP processing, and it is postulated as a neuroinflammation triggering factor. Upregulated P2X7R expression in AD patients and different AD and neuroinflammation mouse models give it a key role in disease progression. Besides, P2X7R also contributes to neuronal loss and synaptic alterations, oxidative stress; inflammasome assembling; and altered microglial function, being all of them processes contributing to AD progression, as described in the present review.

Over the last two decades, several pieces of evidence suggest that some elements of purinergic signaling, in particular P2X7R, might contribute to AD pathology (Parvathenani et al., 2003; McLarnon et al., 2006; Ryu and McLarnon, 2008; Sanz et al., 2009; Delarasse et al., 2011; Diaz-Hernandez et al., 2012; Sanz et al., 2014; Martin et al., 2019; Martinez-Frailes et al., 2019). The first observation of the possible involvement of P2X7R in AD was based on the upregulation of this receptor in microglial cells surrounding senile plaques both in human AD patients and animal models mimicking AD (Parvathenani et al., 2003; McLarnon et al., 2006; Ryu and McLarnon, 2008). Genetic evidence also provided an association between P2X7R and AD, finding a negative correlation among P2X7R 489C>T polymorphism and AD (Sanz et al., 2014). Later studies supplied additional proofs supporting the involvement of P2X7R in the amyloidogenic APP processing (Delarasse et al., 2011; Diaz-Hernandez et al., 2012), synaptic dysfunction (Lee et al., 2011; Saez-Orellana et al., 2016, 2018; Goncalves et al., 2019), oxidative stress (Parvathenani et al., 2003; Lee et al., 2011; Zhang et al., 2015), and neuroinflammation (Kim et al., 2007; Sanz et al., 2009; Chiozzi et al., 2019; Martin et al., 2019; Martinez-Frailes et al., 2019), associated to AD (Figure 1). Supporting the potential therapeutic role of P2X7R, others groups demonstrated that its pharmacological blockade or genetic depletion leads to a significant improvement both symptomatology and neuropathology in AD animal models (Ryu and McLarnon, 2008; Diaz-Hernandez et al., 2012; Chen et al., 2014; Martin et al., 2019). In this review, we are going to examine the different findings supporting a critical role of P2X7R in the regulation of molecular mechanisms underlying AD.

## P2X7R

P2X receptors are plasma membrane ligand-gated ion channels, whose activation causes a selective influx of small cations (Na^+^, Ca^2+^) and K^+^ efflux from cells (Nicke et al., 1998; Hatorri and Gouaux, 2012; Samways et al., 2014). Similar to other members of P2X family, P2X7 subunit has two transmembrane domains linked by a large extracellular domain (Nicke et al., 1998). These subunits present a dolphin-shaped structure with the transmembrane helices and the extracellular region similar to the tail and the body, respectively (McCarthy et al., 2019). Among all P2X receptors, P2X7 is the only one found as a homotrimer in physiological models. Human P2X7R protein is a 595 amino acid protein encoded by the *P2RX7* gene located on chromosome 12 (12q24.31 locus) spanning 53,733 bases (NCBI, 2017). Alternative splicing takes place during gene’s transcription and may give rise to at least 11 different splice variants of P2X7R, described to date (Rassendren et al., 1997; Cheewatrakoolpong et al., 2005; Skarratt et al., 2005, 2020; Feng et al., 2006; Adinolfi et al., 2010; Sluyter and Stokes, 2011). Specific features of full length P2X7 protein include a large C-terminal domain (Virginio et al., 1999); low sensitivity to its native ligand (ATP) and sensitive to extracellular divalent cations (Surprenant et al., 1996). Different intracellular mediators have been associated with P2X7R activation such as calcium calmodulin kinases II (Diaz-Hernandez et al., 2008), nuclear factor kappa-light-chain-enhancer (NFκB) (Ferrari et al., 1997), ROS/NOS formation (Hewinson and MacKenzie, 2007), glycogen synthase kinase-3 (GSK3) (Diaz-Hernandez et al., 2008), phospholipase D (Humphreys and Dubyak, 1996), inflammasome “NACHT, LRR, and PYD domains-containing protein 3” (NLRP3) (Franceschini et al., 2015). However, sustained activation of P2X7R by high ATP concentrations may induce apoptosis or necrosis in some cellular lineages (Virginio et al., 1999; Delarasse et al., 2009).

Although its specific distribution in the CNS remains debated (Miras-Portugal et al., 2017; Illes et al., 2017), P2X7R expression has been reported in almost all cellular lineages making up the brain tissue, including astrocytes, microglia, oligodendrocytes, and neurons (Matute et al., 2007; Miras-Portugal et al., 2017). Interestingly, P2X7R has also been related to several physiological events including neuronal differentiation (Messemer et al., 2013; Tsao et al., 2013; Glaser et al., 2014; Fumagalli et al., 2017), axonal growth and branching (Diaz-Hernandez et al., 2008), presynaptic regulation and neurotransmitter release (Sperlagh et al., 2002; Miras-Portugal et al., 2003; Leon et al., 2008), microglial activation, migration, and proliferation (Sanz et al., 2009; Rigato et al., 2012; Martinez-Frailes et al., 2019), glial and microglial phagocytosis (Ni et al., 2013; Gu and Wiley, 2018; Martinez-Frailes et al., 2019).

### Upregulation of P2X7R in Alzheimer’s Disease

One of the initial pieces of evidence suggesting a possible involvement of P2X7R in AD was the increased P2X7R expression found in microglial cells surrounding amyloid plaques both in AD patients and different AD mouse models (Parvathenani et al., 2003; McLarnon et al., 2006). Later studies using two different mouse models of AD based on the transgenic expression of human APP (APP/PS1 mice and J20 mice, thoroughly described below) confirmed that P2X7R upregulation in activated microglial was parallel with AD progression (Lee et al., 2011; Martinez-Frailes et al., 2019). Another study showed that 9-months-old P301S tau mice, overexpressing mutant human protein tau (MAPT P301S) driven by the mouse prion protein (*Prnp*) promoter (Yoshiyama et al., 2007), show a higher cerebral binding of a radiotracer for P2X7R, [^123^I]TZ26019, than their corresponding WT control mice. Subsequent analysis revealed that P2X7R was mainly found in hippocampal astrocytes in P301S mice (Jin et al., 2018). However, no robust data about the molecular mechanism causing the glial P2X7R upregulation were provided in these studies. Although astroglial activation is a histopathological mark associated with tau-induced toxicity, including AD, little is know about how this cellular type contributes to tau-induced toxicity (Forrest et al., 2019). Considering the critical role of P2X7R on the astrocytic function (Miras-Portugal et al., 2017), additional studies to determinate whether astroglial P2X7R is contributing to tau-associated pathology should be done.

A recent study reported that PS2 deficient mice are most sensitive to Aβ-induced neuroinflammation due to the upregulation of P2X7R in both glial and neuronal cells in a transcription factor Sp1 (SP1)-dependent manner (Qin et al., 2017). Taking into account that SP1 is a transcription factor promoting P2X7R expression (Garcia-Huerta et al., 2012) and that neuroinflammation causes SP1 upregulation via activation of intracellular kinases cascades (Citron et al., 2008), it is reasonable to think that dysregulation of SP1 may be the factor causing P2X7R upregulation detected in AD. Supporting the involvement of SP1 in the molecular mechanisms underlying Aβ-induced toxicity in AD, a significant increase in both SP1 messenger and protein levels was found in the cortex of AD patients and in two mouse models of AD, APP/PS1 mice and Tg2576 mice (Citron et al., 2008). These mice overexpress human APP containing the Swedish mutation under the control of the hamster prion protein promoter (Hsiao et al., 1996). In this line, it was also reported that SP1 as a transcription factor is able to regulate the expression of both APP and tau proteins (Izumi et al., 1992; Heicklen-Klein and Ginzburg, 2000). However, the potential role of SP1 as a therapeutic target in AD was put in doubt after the observation that its sustained pharmacological inhibition, induced by a selective SP1 inhibitor (mithramycin), caused a significative memory deficit and increased the Aβ_1__–__42_ and Aβ_1__–__40_ ratio (Citron et al., 2015). These results indicate that global inhibition of SP1 contributes to neurodegeneration rather than plays a protective role. These side effects could be due to the wide range of different off-target genes regulated by SP1 transcription factor (Bird, 1986; Larsen et al., 1992; Siegfried et al., 1999).

### P2X7R in Amyloidogenic APP Processing

Longitudinal studies combining cognitive assessment, PET analysis, and the measurement of pathognostic molecules from the CSF of eFAD and late-onset AD patients showed an early deposition of Aβ in the precuneus and other cortical areas 10–12 years before first AD symptoms appear (Vermunt et al., 2019). Based on these findings, it is currently accepted that Aβ accumulation represents the initial event triggering the disease. Preceding the onset of cognitive impairment, following the initial Aβ accumulation, a sequential tau accumulation can be observed (Morris et al., 2009; Bateman et al., 2012; Fagan et al., 2014; Gordon et al., 2018; Hanseeuw et al., 2019). However, the existence of cases in which senile plaque burden was detected in brains collected from healthy individuals with no dementia (Shankar et al., 2008; Perez-Nievas et al., 2013), put in doubt that Aβ is the only factor triggering AD. As described above, although APP protein may be processed by both amyloidogenic and non-amyloidogenic pathways in the CNS (Hardy and Selkoe, 2002), it is postulated that in healthy brains APP is preferably cleaved via the non-amyloidogenic pathway (Tyler et al., 2002). Nevertheless, deregulation in this balance might favor amyloidogenic processing, leading to Aβ accumulation (Stockley and O’Neill, 2008).

Different studies using both *in vitro* and *in vivo* approaches postulated that P2X7R might be one of the factors controlling APP processing (Delarasse et al., 2011; Leon-Otegui et al., 2011; Darmellah et al., 2012; Diaz-Hernandez et al., 2012). APP protein can be processed in two different ways. The amyloidogenic pathway is mediated by β- and γ-secretase and results in the generation of extracellular sAPPβ, Aβ-peptides, and the intracellular C-terminal fragment C99. On the other hand, the non-amyloidogenic pathway involves the α-and γ-secretases and results in the generation of an intracellular C-terminal fragment, called C88, extracellular peptides sAPP and the P3 peptides (Selkoe, 2001). Preliminary studies, using mouse neuroblastoma cells (N2a) expressing human APP, reported that BzATP-induced P2X7R activation stimulates the release of sAPP in a mitogen-activated protein kinases (MAPK)-dependent manner. This release was inhibited by selective P2X7R knock-down with siRNA and by specific P2X7R antagonists (Delarasse et al., 2011). In a subsequent study, this group reported that Ezrin/Radixin/Moesin (ERM) are required for P2X7R-dependent processing of APP (Darmellah et al., 2012). However, another group, using two different cell lines, found that the inhibition and not the activation of native or overexpressed P2X7R increases α-secretase activity (Leon-Otegui et al., 2011; Diaz-Hernandez et al., 2012). *In vivo* studies confirmed that pharmacological blockade of P2X7R reduces size and number of hippocampal senile-plaques in 8-months-old J20 mice (PDGF-APPSw,Ind). These mice overexpress human APP with the Swedish (APP KM670/671NL) and Indiana (APP V717F) mutations under the control of platelet-derived growth factor subunit B (PDGFB) promoter (Mucke et al., 2000). This beneficial effect was GSK3-dependent (Diaz-Hernandez et al., 2012). To shed light on these contradictory results, a recent study generated P2X7R deficient APP/PS1 mice. APP/PS1 mice express a chimeric mouse/human APP and human PSN1, with the deletion of exon 9 found in eFAD patients, under the mouse prion protein promoter (APP/PSN1/P2X7^–/–^) (Jankowsky et al., 2004). Results obtained in this study confirmed that genetic depletion of P2X7R leads to a significant reduction in the number of senile plaques in 10-months-old APP/PS1 mice. This decrease was accompanied by a drastic decreasing in Aβ peptides levels and rescue of the cognitive deficit developed by APP/PS1 mice (Martin et al., 2019). All previous data suggest that dysregulation of P2X7R signaling may be one of the factors promoting APP amyloid processing in AD.

### P2X7R in Neuroinflammation Associated With AD

It is widely known that accumulation of Aβ in senile plaques initiates the inflammatory process on AD (McGeer et al., 2000) and favors the activation of the microglial cells around them (Selkoe, 2002). In these cells, the P2X7R upregulation suggests its involvement in microglia cells mediated-neuroinflammatory response on AD (Parvathenani et al., 2003; McLarnon et al., 2006). In the following sections, we will describe how the upregulation of P2X7R in microglial cells contributes to neuroinflammation and how this impacts on microglial functionality.

#### P2X7R in Inflammasome Activation

Microglial cells have dual effects on AD progression. On one side, they promote a decrease in A*β* accumulation by stimulating its phagocytosis, clearance, and degradation. On the other side, chronic microglial activation leads to the release of pro-inflammatory cytokines that can contribute to the neuronal loss (Wang S. et al., 2015; Wang W. Y. et al., 2015). This dual effect may be caused by the activation of microglial cells in two subsets that present different molecular phenotype: the classical (M1) or the selective (M2) activated state (Czeh et al., 2011; Thawkar and Kaur, 2019). M1 state-activated microglia cells promote the release of pro-inflammatory cytokines, playing a pivotal role in the defense against pathogens or tumor cells. M2 state-activated microglia cells secrete anti-inflammatory cytokines promoting tissue repairment (McGeer et al., 2000; Walker and Lue, 2015; Wang W. Y. et al., 2015). However, this is a simplified classification since and microglia cells may acquire other activation states (Ransohoff, 2016). At this regard, a recent genome-wide transcriptome analysis of microglia from models of different neurodegenerative disease has allowed to identify a new disease-associated microglia (DAM) phenotype (Krasemann et al., 2017; Song and Colonna, 2018). DAM, in addition, to express genes characteristic in both classical M1 macrophages and classical M2 macrophages, also express others related to the interferon response, stress response, lysosomal function, and lipid metabolism (Song and Colonna, 2018). One of the first molecules directly linked to activation of DAM was TREM2, a single-immunoglobulin-domain-containing macrophage-specific receptor. Interestingly, recent studies have postulated that this protein plays a central role in the onset and the development of AD because microglia surround and enclose neuritic plaques in a TREM2-dependent manner (Wang Y. et al., 2015; Meilandt et al., 2020).

Data obtained from studies using lipopolysaccharide (LPS)-induced neuroinflammation animal model has contributed clarify the role of microglial cells in neuroinflammation (Boche et al., 2013). LPS activates Toll-like receptor 4 (TLR4) after binding LBP (LPS-binding protein), an intracellular signaling pathway that leads to the activation of nuclear factor NF-kB by a Myeloid differentiation primary response protein MyD88 (MyD88)-dependent mechanism. When activated, NF-kB translocates to the nucleus, binding the DNA, and promoting the transcription of proinflammatory mediators, such as proinflammatory cytokines like pro-interleukin-1 beta (pro-IL1β), pro-interleukin-18 (pro-IL18), and NLRP3 inflammasome (Takeda and Akira, 2004; Venigalla et al., 2016). Activation of this intracellular pathway is a priming event. However, to trigger NLRP3 inflammasome, a cytosolic multiprotein oligomer responsible for the activation of inflammatory responses, a subsequent signal causing a decrease K^+^ levels in the cytosolic microenvironment is required to facilitate the oligomerization of NLRP3. Afterward, the inflammasome recruits the apoptosis-associated speck-like protein (ASC) and the procaspase-1 (another apoptosis-related protein), leading to the secretion of IL1β and IL18 (Petrilli et al., 2007; He et al., 2016). Several studies have shown that ATP, found at high extracellular concentrations following insults (Burnstock, 2008, 2016) or released by Aβ peptide (Kim et al., 2007; Sanz et al., 2009; Saez-Orellana et al., 2018; Goncalves et al., 2019), may be one of the signals promoting NLRP-inflammasome assembling and subsequent IL1β processing (Laliberte et al., 1999; Perregaux et al., 2000; Ye et al., 2013). Interestingly, recent studies have reported that NLRP3 activation may also induce tau hyperphosphorylation and aggregation in an IL-1β-dependent manner (Ising et al., 2019). Initially, *in vitro* studies using microglial cells isolated from rat brains showed that fibrillar Aβ_1__–__42_ peptide-induced ATP-release by P2X7R-dependent mechanism (Kim et al., 2007). Later, using both *in vitro* and *in vivo* approaches, it was suggested that Aβ-induced microglial activation requires the activation of P2X7R via an autocrine/paracrine stimulatory loop (Sanz et al., 2009). Additional research revealed that Aβ causes via a P2X7R-dependent mechanism NF-kB activation and NLRP3 inflammasome expression in microglial cells (Chiozzi et al., 2019). Since pretreatment of cultured microglial cells with potassium chloride (KCl), avoided microglial NRLP3 activation (Gustin et al., 2015), it is reasonable to postulate that efflux of K^+^ induced by P2X7R activation is the mechanism by which P2X7R promotes NRLP3 activation in microglial cells. These studies are suggesting that P2X7R/NLRP3/Caspase1 signaling is a crucial pathway in the inflammasome activation once the microglial cell is primed. In accordance with this hypothesis, Martinez-Frailes et al. (2019) found that upregulation of P2X7R in microglial cells takes place in advanced and late stages of AD, but not in the early stages, when the microglial priming has not yet occurred, and there is a reduced number of senile plaques. In line with this concept, inflammasome activation in APP/PS1 mice occurs in an age- and Aβ deposition-related fashion (Heneka et al., 2013). Recent studies have also proposed the involvement of P2X7R in the initial microglial priming induced by serum amyloid A (SAA) protein via TLR4 activation (Niemi et al., 2011; Facci et al., 2018). SAA is a high-density apolipoprotein generated in the liver and released to the systemic circulation, where it is mainly found associated with HDL, in response to inflammation, reaching different organs including the brain (Gabay and Kushner, 1999). In AD patients, SAA co-localized with cerebral amyloid Aβ-peptide deposits (Kindy et al., 1999) and it is present in high levels in CSF of AD patients (Miida et al., 2006). In cortical microglial cells isolated from rat brain, it was reported that SAA causes microglial priming and inflammasome activation in a P2X7R-dependent manner (Facci et al., 2018).

As, in recent years, it has been suggested that the control or reduction of the chronic neuroinflammation associated with neurodegenerative diseases may be an efficient therapeutic strategy (Beamer et al., 2016; Ribeiro et al., 2019; Thawkar and Kaur, 2019). Therefore, the regulation of neuroinflammation associated with AD by P2X7R is gaining relevance as a possible remedial to fight these disorders. This affirmation is based on the concept that chronic neuroinflammation may contribute to neurodegeneration by promoting the release of proinflammatory cytokines, increasing the permeability of blood–brain barrier (BBB) favoring the recruitment of systemic immune effectors cells and causing a synaptic dysfunction leading to neuronal loss (O’Callaghan et al., 2008; Thawkar and Kaur, 2019). In agreement with this, it has been reported that pharmacological blockade or knocking out the P2X7R in different AD mouse models have positive effects by reducing neuroinflammation (Ryu and McLarnon, 2008; Chen et al., 2018; Martin et al., 2019). Initial studies reported that *in vivo* pharmacological inhibition of P2X7R by Brilliant Blue G (BBG) attenuated inflammatory response and diminished leakiness of BBB induced by intracerebroventricular (i.c.v.) injection of Aβ_1__–__42_ peptide in rat hippocampus (Ryu and McLarnon, 2008). In accordance, later study revealed that *in vivo* inhibition of P2X7R by i.p. administration of BBG prevented the spatial memory impairment and cognitive deficiency in an AD mouse model (Chen et al., 2014). Another study reported that i.c.v. administration of oxidized ATP (o-ATP), a P2X7R antagonist, attenuated microglial activation and neuronal damage induced by i.c.v. administration of LPS (Choi et al., 2007). On the other hand, a sustained P2X7R inhibition by BBG did not modify either the number or the morphology of astroglia or microglial cells. Although, the specific treatment, did reduce IL1β secretion and promote the non-amyloidogenic APP processing in the hippocampus of young J20 mice (Diaz-Hernandez et al., 2012). Moreover, a recent study has reported that APP/PS1/P2X7R deficient mice present smaller cognitive deficit and better synaptic plasticity than APP/PS1 mice. Furthermore, knocking out P2X7R reduces Aβ-induced chemokines release in glial cells, especially C-C motif chemokine 3 (CCL3), which is related to the pathogenic CD8^+^ T-cells recruitment (Martin et al., 2019). All these studies suggest that BBB permeable compounds and selective P2X7R antagonists might be considered as good therapeutic drugs to treat chronic neuroinflammation associated with AD.

#### P2X7R in Microglial Migration

During neuroinflammation, extracellular ATP and other nucleotides seem to act as “find me” and “eat me” signals (Di Virgilio et al., 2009). This hypothesis is based on the fact that extracellular ATP is capable of inducing morphological changes in microglial cells favoring their rapid migration toward local brain injury (Davalos et al., 2005). Initial studies indicated that extracellular purines modulate the microglial migration through their specific metabotropic receptors P2Y12, P2Y1, or P2Y6 (Inoue, 2008; De Simone et al., 2010; Bernier et al., 2013; Langfelder et al., 2015). However, new findings also suggest the involvement of ionotropic purinergic receptors in this phenomenon (Martinez-Frailes et al., 2019). Using *in vitro* and *in vivo* approaches, Martinez-Frailes et al. (2019) have recently confirmed that ATP-induced P2X7R activation promotes microglial migration. These findings might explain why there is an enrichment on P2X7R positive microglial cells around the senile plaques both in AD mouse models and in postmortem brain samples from AD patients (Parvathenani et al., 2003; McLarnon et al., 2006; Lee et al., 2011; Martinez-Frailes et al., 2019). In this line, it reported that P2X7R upregulation in the microglial cell increases in parallel to the incidence of senile plaques (Lee et al., 2011). Moreover, *in vivo* blockade of P2X7R by BBG also caused a reduction of GSK3 activity in P2X7R-expressing microglial cells, by increasing p-GSK3 levels (Diaz-Hernandez et al., 2012). Other *in vitro* studies using BV-2 mouse microglial cells or brain slices also confirmed that GSK3 inhibitors significantly reduced the migratory capacity of microglial cells (Yuskaitis and Jope, 2009). All these findings suggest that P2X7R activation not only promotes NLRP3 inflammasome assembling but also stimulates microglial migration.

#### P2X7R in Microglial Phagocytosis

Another microglia feature that has been related to purinergic signaling is its phagocytic capacity. First studies suggested that purinergic compounds modulated the phagocytic capacity of microglial cells through the metabotropic P2Y6 receptor (Inoue, 2008). However, recent studies have provided additional data indicating that other purinergic receptors may also regulate this microglial function, as P2X7R. In this line, *in vitro* studies using mouse primary microglial cells isolated from the hippocampus demonstrated that both P2X7R genetic depletion using specific RNAi and its pharmacological inhibition by BBG favors microglial phagocytosis of fibrillar Aβ_1__–__42_ and decreases IL1β secretion capacity. In this line, the enrichment of the culture medium with IL1β reduced the microglial Aβ_1__–__42_ phagocytosis (Ni et al., 2013). Additional evidence on P2X7R mediated-regulation of microglial phagocytosis has also been provided by later studies (Janks et al., 2018; Martinez-Frailes et al., 2019). Using both *in vitro* and *in vivo* approaches, it has been found that GSK 1482160A, a selective P2X7R inhibitor, significantly increased the phagocytosis of 2 μm diameter fluorescence microspheres by microglial cells expressing P2X7R (Martinez-Frailes et al., 2019). Accordingly, other *in vitro* studies using cultured primary human microglial cells confirmed that P2X7R activation induced by 300 μM Bz-ATP decreased the phagocytic capacity of microglial cells. That effect was prevented when they used the selective P2X7R antagonist 50 μM A438079 but not by the selective P2X4R antagonist Bx430 (Janks et al., 2018). In agreement with these findings, ATP induced P2X7R activation causes cytoskeleton changes in microglial, reducing their phagocytic capacity (Fang et al., 2009). In this line, and taking into account the important role that microglial phagocytosis plays in the removing of senile plaques, it is to worth highlighting that, both in the hippocampus of J20 mice and in postmortem cortical samples from human AD patients, the majority of microglial cells in contact with senile plaques did not express P2X7R. Furthermore, the percentage of microglia expressing P2X7R inside extracellular Aβ deposits remains constant along the AD progression, in opposition to the rising of the total number of microglial cells (Martinez-Frailes et al., 2019). Interestingly, in human microglial cells, BzATP-induced P2X7R activation reduced its phagocytic capacity and produced mature caspase-1 by activating the inflammasome, revealing a close relationship between both events (Janks et al., 2018). Supporting this concept, the NLRP3 inflammasome inhibitor, MCC950, stimulates Aβ phagocytosis *in vitro* and reduces the number of senile hippocampal plaques in APP/PS1 mice, causing an improvement in their cognitive function (Dempsey et al., 2017). Indeed, double transgenic mice resulting from the crossbreed of APP/PS1 mice and NLRP3^–/–^ or caspase-1^–/–^ (CASP1) mice exhibited a significant reduction in the loss of spatial memory and an enhanced Aβ clearance, compared with APP/PS1 mice. Furthermore, the NLRP3 inflammasome deficiency promoted the switch of microglial cells to M2 state, resulting in decreased deposition of Aβ in APP/PS1 mice (Heneka et al., 2013). This evidence strongly suggests that therapeutic strategies focusing either on direct inflammasome inhibition or on avoiding its assembling by blocking P2X7R, might be efficient for reducing neuroinflammation and promoting Aβ phagocytosis in AD.

### P2X7R in Oxidative Stress Associated With AD

Oxidative stress is a condition where the generation of reactive oxygen species (ROS) exceeds the capacity of antioxidative mechanisms of the cell (Zuo et al., 2015). High levels of ROS are commonly detected in the brain of patients suffering from different neurodegenerative diseases, including AD (Albers and Beal, 2000; Sebastian-Serrano et al., 2019). Although these species cannot trigger the neurodegenerative disease on their own, they might favor its progression by promoting the oxidative damage and interacting with mitochondria (Dias et al., 2013). Thereby, accumulation of ROS might cause mitochondrial alterations, resulting in increased ROS production and consequent mitochondrial dysfunction, favoring the disease progression. Accordingly, the content of ATP in the tissue is reduced in parallel to the disease progression in APP/PS1 mice. This fact is suggesting that this decline is caused by mitochondrial dysfunction provoked by ROS accumulation (Zhang et al., 2015). Moreover, Aβ can lead to ROS production, in particular, hydrogen peroxide (H_2_O_2_), that causes the damage of proteins, lipids, and nucleic acids (Eckert et al., 2003). Several pieces of evidence point to the fact that P2X7R may be the primary receptor involved in the generation of H_2_O_2_ by activating microglial cells (Nuttle and Dubyak, 1994). Stimulation of isolated microglial cells from rat brain with ATP or BzATP, induced O_2_^–^ release in NADPH oxidase activation-dependent mechanism. Furthermore, inhibition of phosphatidylinositol 3 kinase, a kinase involved in GSK-3 signaling, attenuated BzATP-induced H_2_O_2_ release, preventing microglial-induced cortical death (Parvathenani et al., 2003). *In vitro* studies reported that fibrillar Aβ_1__–__42_ causes ROS production generated via P2X7R activation induced by ATP released from rat microglial cells in an autocrine manner (Kim et al., 2007; Liu et al., 2020). Additional studies revealed that P2X7R positive microglial cells surrounding senile plaques express the catalytic NADPH subunit (gp91^phox^) and produce ROS species in APP/PS1 mice. Hence, P2X7R upregulation in microglial cells may result in excessive ROS production induced by Aβ via P2X7R, which contributes to the synaptic toxicity associated with the early stages of AD (Lee et al., 2011). Recently, a study using P2X7R-deficient microglial cell line (N13R) has demonstrated that Aβ-induced mitochondrial toxicity requires P2X7R in microglial cells (Chiozzi et al., 2019). In agreement with the antioxidative effect of P2X7R antagonists, *in vivo* administration of selective P2X7R antagonist A438073, avoided ROS production and oxidative DNA damage induced by P2X7R activation in spinal cord dorsal horn neurons (Munoz et al., 2017).

### P2X7R in Synaptic Dysfunction and Cellular Death in AD

Another major hallmark of AD is the extensive loss of synapses correlating with cognitive impairment (Lansbury, 1999). One of the most robust pieces of evidence indicating that Aβ deposits contribute to synaptic loss is the observation that a degree of synaptic loss is more evident in the proximity of the senile plaques (Lanz et al., 2003). Initial studies postulated that the synaptic loss detected in APP/PS1 mice was due to the dysfunction and collapse of the excitatory synapses. This fact was caused by the interaction of the soluble Aβ peptide oligomers coming from the surrounding plaques with these synaptic contacts (Koffie et al., 2009). However, the molecular mechanism by which Aβ alters synaptic transmission causing a subsequent synaptic loss remains unclear (Brody and Strittmatter, 2018). One hypothesis is that Aβ may interact directly with neuronal synaptic receptors such as metabotropic glutamate receptor 5 mGluR5 (Um et al., 2013), or α7 nicotine acetylcholine receptor α7nAChR (Wang et al., 2000). Others postulate that microglial cells activated by Aβ are responsible for assaulting the synapses (Hong et al., 2016). Nevertheless, new evidence suggests that synaptic dysfunction associated with AD may be due to dysregulation of P2X receptors mediated neurotransmission; dysregulation that may be triggered by increased extracellular ATP concentration induced by Aβ (Chen et al., 2014; Saez-Orellana et al., 2016, 2018; Goncalves et al., 2019). Supporting this hypothesis, higher K^+^ depolarization-induced ATP release was found in hippocampal nerve terminals isolated from i.c.v. Aβ_1–42_-treated mice (Goncalves et al., 2019). According to this, pharmacological blockade of P2X7R prevents the increase in the current frequency of the excitatory synapse induced by oligomeric Aβ when binding to excitatory neurons (Saez-Orellana et al., 2016, 2018). Furthermore, pharmacological inhibition of P2X7R prevented Aβ-induced loss of filopodia and spine density in cultured hippocampal neurons (Chen et al., 2018). Other studies have provided additional evidence suggesting that P2X7R-mediated ROS production in Aβ-stimulated microglial is one of the mechanisms explaining oligomeric Aβ-mediated synaptic-toxicity in APP/PS1 mice (Lee et al., 2011). In accordance with this toxic effect, BzATP-induced P2X7R activation caused microglia-induced cortical cell death (Parvathenani et al., 2003). Choi et al. (2014) reported similar results, observing that *in vivo* P2X7R blockade by o-ATP reduces the number of positive caspase-3 neurons in LPS-injected brains (Choi et al., 2007). In concordance with the involvement of P2X7R in the synaptic-toxicity induced by Aβ, P2X7R deficiency rescued the synaptic alterations and LTP deficits detected in APP/PS1 mice (Martin et al., 2019). Interestingly, it was reported that, contrary to what was observed in microglial cells, neuronal P2X7R transcription is reduced in J20 mice both in early and advanced stages. This points to the fact that this phenomenon could be an adaptive physiological response to avoid or at least lessen the neuronal loss associated with AD. However, the loss of this capacity may contribute to the exacerbation of neuronal loss in the late stages of AD (Martinez-Frailes et al., 2019).

## Induced Pluripotent Stem Cells in AD Research

The currently available knowledge regarding AD is mostly based on results acquired from post-mortem patient samples or animal models mimicking the disease. However, because human brain tissue is extremely hard to obtain, especially if there is a need for early-onset materials, a need for a human-derived *in vitro* system arose. Thus, the Nobel Prize awarded induced pluripotent stem cell (iPSC) technology that allows the genetic reprogramming of mature somatic cells into pluripotent stem cells (PSCs) (Takahashi et al., 2007) became widely utilized.

The differentiation of cells from patient-specific iPSCs provides valuable insight into specific molecular phenotypes of neurodegenerative diseases (McKinney, 2017) because the cells possess the complete genetic background of the patient. Moreover, healthy individuals’ derived iPSCs can be genetically modified to introduce disease-specific genetic patterns (Ortiz-Virumbrales et al., 2017).

Many neurodegenerative disease models are available to date. For example, AD patient-derived iPSCs are being used by many research groups (Israel et al., 2012; Kondo et al., 2017; Ochalek et al., 2017; Sullivan and Young-Pearse, 2017; Arber et al., 2019; Chang et al., 2019).

Focusing on AD research, the use of iPSC-derived cells has helped to discover new pathological mechanisms underlying AD pathology. For instance, describing for the first time an autophagic dysfunction due to lysosomal depletion and suggesting that modifying the lysosomal biogenesis could present a novel therapeutic intervention (Lee et al., 2014). Besides, iPSC-derived cells responded very differently to drug treatments than APP-overexpressing cell lines and thus demonstrated that it can be a better option for preclinical screening of compounds (Liu et al., 2014). iPSC-models have helped to prove that β-secretase has a higher affinity for neuregulin (NRG1) than for APP, which means that it might be possible to inhibit Aβ production via BACE1 processing without affecting BACE1 interactions with its other substrates (Ben Halima et al., 2016). Importantly, iPSC-based systems are suitable for compound screening. A correlation between CSF profiles from patients and their own Aβ secretome in the differentiated neuronal cultures was found, showing the relevance of iPSC derived systems in AD modeling (Kondo et al., 2017). In AD iPSC-derived neurons, constitutional metabolic changes in ROS production without mitochondrial fission and fusion proteins damage have been described. These findings suggest that increased ROS production might have a more important role in amyloid- and tau-pathology than previously anticipated (Birnbaum et al., 2018). Furthermore, findings in these models shown tau protein species propagation patterns where tau oligomers, but not monomers, induced accumulation of pathological, hyperphosphorylated tau in human neurons (Usenovic et al., 2015). Therefore, it is realistic to expect that this technology will provide valuable insights into the P2X7R research field in the near future.

Application of 3D cell cultures will help to model more reliably the brain tissue cell interactions and microenvironment, including gradients of signaling molecules (Zhang et al., 2014). Particularly for neurodegeneration research, 3D systems promote the formation of specific neuronal cell types with complex interactions and development of AD pathologies, taking into account gradients of signaling molecules such as ATP and the subsequent cellular responses within the tissue (Mungenast et al., 2016).

## Concluding Remarks

Due to the fact that many anti-amyloid clinical trials have failed, Aβ-directed therapies focusing on the reduction of parenchymal Aβ and amyloid deposits in AD brains have been put in doubt (Long and Holtzman, 2019). Many strategies were tested: active and passive immunization, secretase inhibitors or drugs avoiding amyloid aggregation, but none of them was effective in modifying the disease course in symptomatic AD patients (Hara et al., 2019). However, there are still ongoing active Phase III clinical trials based on monoclonal antibodies against Aβ, new anti-inflammatory molecules and to induce an active immunization, whose results could be concluded later (Long and Holtzman, 2019). As results from studies using animal models, mimicking AD pathology are strongly suggesting that Aβ is the triggering disease factor, perhaps, amyloid-direct therapies would be more useful to treat preclinical cases. Moreover, the vast number of symptomatic AD patients require the urgent development of new therapeutic strategies. In recent years, strategies focused on modulating neuroinflammation, or microglial response have taken strength. So, taking this into account, selective P2X7R antagonists might be considered as potential therapeutic drugs. In addition to reducing the Aβ burden, promoting the non-amyloidogenic APP and processing, P2X7R antagonists have also shown anti-inflammatory, neuroprotective, and antioxidant effects, which might counter the pathological conditions associated with AD. Although human iPSC-based studies have not yet reported on the expression of P2X7R in the in vitro models, either on iPSC-derived neurons or astrocytes, the increasing number of AD patient-derived iPSC disease models will promote the emergence of such investigations toward potential therapeutic targets. Indeed, taking into account the complex pathological state found in AD and other neurodegenerative diseases, nowadays it is postulated that many factors together play a role in facilitating the progressive and detrimental neurodegenerative process. Since major biological systems of the human body are involved—such as the nervous system itself, the immune system, the endocrine system, possibly the digestive system (Jiang et al., 2017; Vogt et al., 2017) and perhaps others, currently unknown features and mechanisms, it is extraordinarily challenging to target only one of the systems or pathological processes in the attempt to cure neurodegenerative diseases. Perhaps the time has come to rethink the therapeutic strategies to treat these diseases, in a way where multiple mechanisms could be pharmacologically targeted at the same time, as long as the individual interventions could add up and lead to the elimination of the progression or even the symptoms of dementia. As we discussed in this review, P2X7R may be an excellent target for this multi-target therapy.

## Author Contributions

MD-H contributed to conceptualization and writing. MD-H, LF, and CB contributed to original draft preparation. CD, ÁS-S, LD-G, JK, and AD contributed to writing – review and editing. All authors read and approved the final version of the manuscript.

## Conflict of Interest

LF, JK, and AD are employed by the company BioTalentum Ltd. The remaining authors declare that the research was conducted in the absence of any commercial or financial relationships that could be construed as a potential conflict of interest.

## References

[B1] Adinolfi E. Cirillo M. Woltersdorf R. Falzoni S. Chiozzi P. Pellegatti P. (2010). Trophic activity of a naturally occurring truncated isoform of the P2X7 receptor. *FASEB J.* 24 3393–3404. 10.1096/fj.09-153601 20453110

[B2] Aisen P. S. Davis K. L. Berg J. D. Schafer K. Campbell K. Thomas R. G. (2000). A randomized controlled trial of prednisone in Alzheimer’s disease. Alzheimer’s disease cooperative study. *Neurology* 54 588–593. 10.1212/wnl.54.3.588 10680787

[B3] Alam R. Driver D. Wu S. Lozano E. Key S. L. Hole J. T. (2017). Preclinical characterization of an antibody [Ly3303560] Targeting Aggregated Tau. *Alzheimers Dement.* 13 592–593. 10.1016/j.jalz.2017.07.22728238739

[B4] Albers D. S. Beal M. F. (2000). Mitochondrial dysfunction and oxidative stress in aging and neurodegenerative disease. *J. Neural Transm. Suppl.* 59 133–154. 10.1007/978-3-7091-6781-6_16 10961426

[B5] Alzforum (2018). *RE: To Block Tau’s Proteopathic Spread, Antibody Must Attack its Mid-Region.* Available online at: https://www.alzforum.org/news/conference-coverage/block-taus-proteopathic-spread-antibody-must-attack-its-mid-region (accessed December 9, 2019).

[B6] Alzforum (2019). *Therapeutics Search | ALZFORUM.* Cambridge, MA: Alzheimer Research Forum.

[B7] Alzheimer’s-Association (2019). 2019 Alzheimer’s disease facts and figures. *Alzheimers Dement.* 15 321–387. 10.1016/j.jalz.2019.01.010

[B8] Arber C. Toombs J. Lovejoy C. C. Ryan N. S. Paterson R. W. Willumsen N. (2019). Familial Alzheimer’s disease patient-derived neurons reveal distinct mutation-specific effects on amyloid beta. *Mol. Psychiatry.* 10.1038/s41380-019-0410-8 [Epub ahead of print]. 30980041 PMC7577860

[B9] Avila J. (2006). Tau protein, the main component of paired helical filaments. *J. Alzheimers. Dis.* 9(3 Suppl.), 171–175. 10.3233/jad-2006-9s320 16914856

[B10] Bateman R. J. Xiong C. Benzinger T. L. Fagan A. M. Goate A. Fox N. C. (2012). Clinical and biomarker changes in dominantly inherited Alzheimer’s disease. *N. Engl. J. Med.* 367 795–804. 10.1056/NEJMoa1202753 22784036 PMC3474597

[B11] Beamer E. Goloncser F. Horvath G. Beko K. Otrokocsi L. Kovanyi B. (2016). Purinergic mechanisms in neuroinflammation: an update from molecules to behavior. *Neuropharmacology* 104 94–104. 10.1016/j.neuropharm.2015.09.019 26384652

[B12] Ben Halima S. Mishra S. Raja K. M. P. Willem M. Baici A. Simons K. (2016). Specific inhibition of β-secretase processing of the alzheimer disease amyloid precursor protein. *Cell Rep.* 14 2127–2141. 10.1016/j.celrep.2016.01.076 26923602

[B13] Bernier L. P. Ase A. R. Boué-Grabot É. Séguéla P. (2013). Inhibition of P2X4 function by P2Y6 UDP receptors in microglia. *Glia* 61 2038–2049. 10.1002/glia.22574 24123515

[B14] Bird A. P. (1986). CpG-rich islands and the function of DNA methylation. *Nature* 321 209–213. 10.1038/321209a0 2423876

[B15] Birnbaum J. H. Wanner D. Gietl A. F. Saake A. Kündig T. M. Hock C. (2018). Oxidative stress and altered mitochondrial protein expression in the absence of amyloid-β and tau pathology in iPSC-derived neurons from sporadic Alzheimer’s disease patients. *Stem Cell Res.* 27 121–130. 10.1016/j.scr.2018.01.019 29414602

[B16] Boche D. Perry V. H. Nicoll J. A. (2013). Review: activation patterns of microglia and their identification in the human brain. *Neuropathol. Appl. Neurobiol.* 39 3–18. 10.1111/nan.12011 23252647

[B17] Boxer A. L. Qureshi I. Ahlijanian M. Grundman M. Golbe L. I. Litvan I. (2019). Safety of the tau-directed monoclonal antibody BIIB092 in progressive supranuclear palsy: a randomised, placebo-controlled, multiple ascending dose phase 1b trial. *Lancet Neurol.* 18 549–558. 10.1016/S1474-4422(19)30139-5 31122495

[B18] Bridel C. van Wieringen W. N. Zetterberg H. Tijms B. M. Teunissen C. E. the Nfl Group (2019). Diagnostic value of cerebrospinal fluid neurofilament light protein in neurology: a systematic review and meta-analysis. *JAMA Neurol.* 76 1035–1048. 10.1001/jamaneurol.2019.1534 31206160 PMC6580449

[B19] Brody A. H. Strittmatter S. M. (2018). Synaptotoxic signaling by amyloid beta oligomers in Alzheimer’s disease through prion protein and mGluR5. *Adv. Pharmacol.* 82 293–323. 10.1016/bs.apha.2017.09.007 29413525 PMC5835229

[B20] Burnstock G. (2008). Purinergic signalling and disorders of the central nervous system. *Nat. Rev. Drug Discov.* 7 575–590. 10.1038/nrd2605 18591979

[B21] Burnstock G. (2016). An introduction to the roles of purinergic signalling in neurodegeneration, neuroprotection and neuroregeneration. *Neuropharmacology* 104 4–17. 10.1016/j.neuropharm.2015.05.031 26056033

[B22] Carlsson C. M. Gleason C. E. Hess T. M. Moreland K. A. Blazel H. M. Koscik R. L. (2008). Effects of simvastatin on cerebrospinal fluid biomarkers and cognition in middle-aged adults at risk for Alzheimer’s disease. *J. Alzheimers Dis.* 13 187–197. 10.3233/jad-2008-13209 18376061

[B23] Chang K. H. Lee-Chen G. J. Huang C. C. Lin J. L. Chen Y. J. Wei P. C. (2019). Modeling Alzheimer’s disease by induced pluripotent stem cells carrying APP D678H mutation. *Mol. Neurobiol.* 56 3972–3983. 10.1007/s12035-018-1336-x 30238389 PMC6505505

[B24] Cheewatrakoolpong B. Gilchrest H. Anthes J. C. Greenfeder S. (2005). Identification and characterization of splice variants of the human P2X 7 ATP channel. *Biochem. Biophys. Res. Commun.* 332 17–27. 10.1016/j.bbrc.2005.04.087 15896293

[B25] Chen M. Lee H. K. Moo L. Hanlon E. Stein T. Xia W. (2018). Common proteomic profiles of induced pluripotent stem cell-derived three-dimensional neurons and brain tissue from Alzheimer patients. *J. Proteomics* 182 21–33. 10.1016/j.jprot.2018.04.032 29709615 PMC7457321

[B26] Chen X. Hu J. Jiang L. Xu S. Zheng B. Wang C. (2014). Brilliant Blue G improves cognition in an animal model of Alzheimer’s disease and inhibits amyloid-beta-induced loss of filopodia and dendrite spines in hippocampal neurons. *Neuroscience* 279 94–101. 10.1016/j.neuroscience.2014.08.036 25193238

[B27] Chiozzi P. Sarti A. C. Sanz J. M. Giuliani A. L. Adinolfi E. Vultaggio-Poma V. (2019). Amyloid beta-dependent mitochondrial toxicity in mouse microglia requires P2X7 receptor expression and is prevented by nimodipine. *Sci. Rep.* 9:6475. 10.1038/s41598-019-42931-2 31019207 PMC6482182

[B28] Choi H. B. Ryu J. K. Kim S. U. McLarnon J. G. (2007). Modulation of the purinergic P2X7 receptor attenuates lipopolysaccharide-mediated microglial activation and neuronal damage in inflamed brain. *J. Neurosci.* 27 4957–4968. 10.1523/JNEUROSCI.5417-06.2007 17475804 PMC6672082

[B29] Choi S. H. Kim Y. H. Hebisch M. Sliwinski C. Lee S. D’Avanzo C. (2014). A three-dimensional human neural cell culture model of Alzheimer’s disease. *Nature* 515 274–278. 10.1038/nature13800 25307057 PMC4366007

[B30] Citron B. A. Dennis J. S. Zeitlin R. S. Echeverria V. (2008). Transcription factor Sp1 dysregulation in Alzheimer’s disease. *J. Neurosci. Res.* 86 2499–2504. 10.1002/jnr.21695 18449948

[B31] Citron B. A. Saykally J. N. Cao C. Dennis J. S. Runfeldt M. Arendash G. W. (2015). Transcription factor Sp1 inhibition, memory, and cytokines in a mouse model of Alzheimer’s disease. *Am. J. Neurodegener. Dis.* 4 40–48. 26807343 PMC4700125

[B32] Czeh M. Gressens P. Kaindl A. M. (2011). The yin and yang of microglia. *Dev. Neurosci.* 33 199–209. 10.1159/000328989 21757877

[B33] Czerkowicz J. (2017). Pan-Tau antibody Biib076 exhibits promising safety and biomarker profile in Cynomolgus monkey toxicity study. *Alzheimers Dement.* 13 1271–1271.

[B34] Darmellah A. Rayah A. Auger R. Cuif M. H. Prigent M. Arpin M. (2012). Ezrin/radixin/moesin are required for the purinergic P2X7 receptor (P2X7R)-dependent processing of the amyloid precursor protein. *J. Biol. Chem.* 287 34583–34595. 10.1074/jbc.M112.400010 22891241 PMC3464564

[B35] Davalos D. Grutzendler J. Yang G. Kim J. V. Zuo Y. Jung S. (2005). ATP mediates rapid microglial response to local brain injury in vivo. *Nat. Neurosci.* 8 752–758. 10.1038/nn1472 15895084

[B36] de Jong D. Jansen R. Hoefnagels W. Jellesma-Eggenkamp M. Verbeek M. Borm G. (2008). No effect of one-year treatment with indomethacin on Alzheimer’s disease progression: a randomized controlled trial. *PLoS One* 3:e1475. 10.1371/journal.pone.0001475 18213383 PMC2194921

[B37] De Simone R. Niturad C. E. De Nuccio C. Ajmone-Cat M. A. Visentin S. Minghetti L. (2010). TGF-β and LPS modulate ADP-induced migration of microglial cells through P2Y1 and P2Y12 receptor expression. *J. Neurochem.* 115 450–459. 10.1111/j.1471-4159.2010.06937.x 20681951

[B38] Delarasse C. Auger R. Gonnord P. Fontaine B. Kanellopoulos J. M. (2011). The purinergic receptor P2X7 triggers alpha-secretase-dependent processing of the amyloid precursor protein. *J. Biol. Chem.* 286 2596–2606. 10.1074/jbc.M110.200618 21081501 PMC3024755

[B39] Delarasse C. Gonnord P. Galante M. Auger R. Daniel H. Motta I. (2009). Neural progenitor cell death is induced by extracellular ATP via ligation of P2X7 receptor. *J. Neurochem.* 109 846–857. 10.1111/j.1471-4159.2009.06008.x 19250337

[B40] Dempsey C. Rubio Araiz A. Bryson K. J. Finucane O. Larkin C. Mills E. L. (2017). Inhibiting the NLRP3 inflammasome with MCC950 promotes non-phlogistic clearance of amyloid-beta and cognitive function in APP/PS1 mice. *Brain Behav. Immun.* 61 306–316. 10.1016/j.bbi.2016.12.014 28003153

[B41] Di Virgilio F. Ceruti S. Bramanti P. Abbracchio M. P. (2009). Purinergic signalling in inflammation of the central nervous system. *Trends Neurosci.* 32 79–87. 10.1016/j.tins.2008.11.003 19135728

[B42] Dias V. Junn E. Mouradian M. M. (2013). The role of oxidative stress in Parkinson’s disease. *J. Parkinsons Dis.* 3 461–491. 10.3233/JPD-130230 24252804 PMC4135313

[B43] Diaz-Hernandez J. I. Gomez-Villafuertes R. Leon-Otegui M. Hontecillas-Prieto L. Del Puerto A. Trejo J. L. (2012). In vivo P2X7 inhibition reduces amyloid plaques in Alzheimer’s disease through GSK3beta and secretases. *Neurobiol. Aging* 33 1816–1828. 10.1016/j.neurobiolaging.2011.09.040 22048123

[B44] Diaz-Hernandez M. del Puerto A. Diaz-Hernandez J. I. Diez-Zaera M. Lucas J. J. Garrido J. J. (2008). Inhibition of the ATP-gated P2X7 receptor promotes axonal growth and branching in cultured hippocampal neurons. *J. Cell Sci.* 121(Pt 22), 3717–3728. 10.1242/jcs.034082 18987356

[B45] Domínguez J. M. Fuertes A. Orozco L. Monte-Millán M. D. Delgado E. Medina M. (2012). Evidence for irreversible inhibition of glycogen synthase kinase-3β by tideglusib. *J. Biol. Chem.* 287 893–904. 10.1074/jbc.M111.306472 22102280 PMC3256883

[B46] Doody R. S. Raman R. Farlow M. Iwatsubo T. Vellas B. Joffe S. (2013). A phase 3 trial of semagacestat for treatment of Alzheimer’s disease. *N. Engl. J. Med.* 369 341–350. 10.1056/NEJMoa1210951 23883379

[B47] Eckert A. Keil U. Marques C. A. Bonert A. Frey C. Schussel K. (2003). Mitochondrial dysfunction, apoptotic cell death, and Alzheimer’s disease. *Biochem. Pharmacol.* 66 1627–1634. 10.1016/s0006-2952(03)00534-3 14555243

[B48] Egan M. F. Kost J. Tariot P. N. Aisen P. S. Cummings J. L. Vellas B. (2018). Randomized trial of Verubecestat for mild-to-moderate Alzheimer’s disease. *N. Engl. J. Med.* 378 1691–1703. 10.1056/NEJMoa1706441 29719179 PMC6776074

[B49] Egan M. F. Kost J. Voss T. Mukai Y. Aisen P. S. Cummings J. L. (2019). Randomized trial of Verubecestat for prodromal Alzheimer’s disease. *N. Engl. J. Med.* 380 1408–1420. 10.1056/NEJMoa1812840 30970186 PMC6776078

[B50] Facci L. Barbierato M. Zusso M. Skaper S. D. Giusti P. (2018). Serum amyloid A primes microglia for ATP-dependent interleukin-1beta release. *J. Neuroinflammation* 15:164. 10.1186/s12974-018-1205-6 29803222 PMC5970445

[B51] Fagan A. M. Xiong C. Jasielec M. S. Bateman R. J. Goate A. M. Benzinger T. L. (2014). Longitudinal change in CSF biomarkers in autosomal-dominant Alzheimer’s disease. *Sci. Transl. Med.* 6:226ra230. 10.1126/scitranslmed.3007901 24598588 PMC4038930

[B52] Fang K. M. Yang C. S. Sun S. H. Tzeng S. F. (2009). Microglial phagocytosis attenuated by short-term exposure to exogenous ATP through P2X receptor action. *J. Neurochem.* 111 1225–1237. 10.1111/j.1471-4159.2009.06409.x 19860838

[B53] Feldman H. H. Doody R. S. Kivipelto M. Sparks D. L. Waters D. D. Jones R. W. (2010). Randomized controlled trial of atorvastatin in mild to moderate Alzheimer disease: LEADe. *Neurology* 74 956–964. 10.1212/WNL.0b013e3181d6476a 20200346

[B54] Feng Y. H. Li X. Wang L. Zhou L. Gorodeski G. I. (2006). A truncated P2X 7 receptor variant (P2X 7-j) endogenously expressed in cervical cancer cells antagonizes the full-length P2X 7 receptor through hetero-oligomerization. *J. Biol. Chem.* 281 17228–17237. 10.1074/jbc.M602999200 16624800 PMC2409001

[B55] Ferrari D. Wesselborg S. Bauer M. K. A. Schulze-Osthoff K. (1997). Extracellular ATP activates transcription factor NF-κB through the P2Z purinoreceptor by selectively targeting NF-κB p65 (RelA). *J. Cell Biol.* 139 1635–1643. 10.1083/jcb.139.7.1635 9412459 PMC2132650

[B56] Forrest S. L. Kril J. J. Halliday G. M. (2019). Cellular and regional vulnerability in frontotemporal tauopathies. *Acta Neuropathol.* 138 705–727. 10.1007/s00401-019-02035-7 31203391

[B57] Franceschini A. Capece M. Chiozzi P. Falzoni S. Sanz J. M. Sarti A. C. (2015). The P2X7 receptor directly interacts with the NLRP3 inflammasome scaffold protein. *FASEB J.* 29 2450–2461. 10.1096/fj.14-268714 25690658

[B58] Fumagalli M. Lecca D. Abbracchio M. P. Ceruti S. (2017). Pathophysiological role of purines and pyrimidines in neurodevelopment: unveiling new pharmacological approaches to congenital brain diseases. *Front. Pharmacol.* 8:941. 10.3389/fphar.2017.00941 29375373 PMC5770749

[B59] Gabay C. Kushner I. (1999). Acute-phase proteins and other systemic responses to inflammation. *N. Engl. J. Med.* 340 448–454. 10.1056/NEJM199902113400607 9971870

[B60] Gahtan E. Overmier J. B. (1999). Inflammatory pathogenesis in Alzheimer’s disease: biological mechanisms and cognitive sequeli. *Neurosci. Biobehav. Rev.* 23 615–633. 10.1016/s0149-7634(98)00058-x 10392655

[B61] Garcia-Huerta P. Diaz-Hernandez M. Delicado E. G. Pimentel-Santillana M. Miras-Portugal M. T. Gomez-Villafuertes R. (2012). The specificity protein factor Sp1 mediates transcriptional regulation of P2X7 receptors in the nervous system. *J. Biol. Chem.* 287 44628–44644. 10.1074/jbc.M112.390971 23139414 PMC3531778

[B62] Glaser T. de Oliveira S. L. Cheffer A. Beco R. Martins P. Fornazari M. (2014). Modulation of mouse embryonic stem cell proliferation and neural differentiation by the P2X7 receptor. *PLoS One* 9:e96281. 10.1371/journal.pone.0096281 24798220 PMC4010452

[B63] Goncalves F. Q. Lopes J. P. Silva H. B. Lemos C. Silva A. C. Goncalves N. (2019). Synaptic and memory dysfunction in a beta-amyloid model of early Alzheimer’s disease depends on increased formation of ATP-derived extracellular adenosine. *Neurobiol. Dis.* 132:104570. 10.1016/j.nbd.2019.104570 31394204

[B64] Gordon B. A. Blazey T. M. Su Y. Hari-Raj A. Dincer A. Flores S. (2018). Spatial patterns of neuroimaging biomarker change in individuals from families with autosomal dominant Alzheimer’s disease: a longitudinal study. *Lancet Neurol.* 17 241–250. 10.1016/S1474-4422(18)30028-0 29397305 PMC5816717

[B65] Gu B. J. Wiley J. S. (2018). P2X7 as a scavenger receptor for innate phagocytosis in the brain. *Br. J. Pharmacol.* 175 4195–4208. 10.1111/bph.14470 30098011 PMC6193880

[B66] Gustin A. Kirchmeyer M. Koncina E. Felten P. Losciuto S. Heurtaux T. (2015). NLRP3 inflammasome is expressed and functional in mouse brain microglia but not in astrocytes. *PLoS One* 10:e0130624. 10.1371/journal.pone.0130624 26091541 PMC4474809

[B67] Hanseeuw B. J. Betensky R. A. Jacobs H. I. L. Schultz A. P. Sepulcre J. Becker J. A. (2019). Association of Amyloid and Tau with cognition in preclinical Alzheimer disease: a longitudinal study. *JAMA Neurol.* 10.1001/jamaneurol.2019.1424 [Epub ahead of print]. PMC6547132 31157827

[B68] Hara Y. McKeehan N. Fillit H. M. (2019). Translating the biology of aging into novel therapeutics for Alzheimer disease. *Neurology* 92 84–93. 10.1212/WNL.0000000000006745 30530798 PMC6340342

[B69] Hardy J. Selkoe D. J. (2002). The amyloid hypothesis of Alzheimer’s disease: progress and problems on the road to therapeutics. *Science* 297 353–356. 10.1126/science.1072994 12130773

[B70] Hatorri M. Gouaux E. (2012). Molecular mechanism of ATP binding and ion channel activation in P2X receptors. *Nature* 485 207–212. 10.1038/nature11010 22535247 PMC3391165

[B71] He Y. Zeng M. Y. Yang D. Motro B. Nunez G. (2016). NEK7 is an essential mediator of NLRP3 activation downstream of potassium efflux. *Nature* 530 354–357. 10.1038/nature16959 26814970 PMC4810788

[B72] Heicklen-Klein A. Ginzburg I. (2000). Tau promoter confers neuronal specificity and binds Sp1 and AP-2. *J. Neurochem.* 75 1408–1418. 10.1046/j.1471-4159.2000.0751408.x 10987820

[B73] Heneka M. T. Kummer M. P. Stutz A. Delekate A. Schwartz S. Vieira-Saecker A. (2013). NLRP3 is activated in Alzheimer’s disease and contributes to pathology in APP/PS1 mice. *Nature* 493 674–678. 10.1038/nature11729 23254930 PMC3812809

[B74] Henley D. Raghavan N. Sperling R. Aisen P. Raman R. Romano G. (2019). Preliminary results of a trial of Atabecestat in preclinical Alzheimer’s disease. *N. Engl. J. Med.* 380 1483–1485. 10.1056/NEJMc1813435 30970197

[B75] Hewinson J. MacKenzie A. B. (2007). P2X(7) receptor-mediated reactive oxygen and nitrogen species formation: From receptor to generators,” *Biochem. Soc. Trans.* 35(Pt 5), 1168–1170. 10.1042/bst0351168 17956304

[B76] Hong S. Beja-Glasser V. F. Nfonoyim B. M. Frouin A. Li S. Ramakrishnan S. (2016). Complement and microglia mediate early synapse loss in Alzheimer mouse models. *Science* 352 712–716. 10.1126/science.aad8373 27033548 PMC5094372

[B77] Hsiao K. Chapman P. Nilsen S. Eckman C. Harigaya Y. Younkin S. (1996). Correlative memory deficits, Abeta elevation, and amyloid plaques in transgenic mice. *Science* 274 99–102. 10.1126/science.274.5284.99 8810256

[B78] Humphreys B. D. Dubyak G. R. (1996). Induction of the P2z/P2X7 nucleotide receptor and associated phospholipase D activity by lipopolysaccharide and IFN-gamma in the human THP-1 monocytic cell line. *J. Immunol.* 157 5627–5637. 8955215

[B79] Illes P. Khan T. M. Rubini P. (2017). Neuronal P2X7 receptors revised: do they really exist? *J. Neurosci.* 37 7049–7062. 10.1523/jneurosci.3103-16.2017 28747388 PMC6705732

[B80] Inoue K. (2008). Purinergic systems in microglia. *Cell. Mol. Life Sci.* 19 3074–3080. 10.1007/s00018-008-8210-3 18563292 PMC11131657

[B81] Ising C. Venegas C. Zhang S. Scheiblich H. Schmidt S. V. Vieira-Saecker A. (2019). NLRP3 inflammasome activation drives tau pathology. *Nature* 575 669–673. 10.1038/s41586-019-1769-z 31748742 PMC7324015

[B82] Israel M. A. Yuan S. H. Bardy C. Reyna S. M. Mu Y. Herrera C. (2012). Probing sporadic and familial Alzheimer’s disease using induced pluripotent stem cells. *Nature* 482 216–220. 10.1038/nature10821 22278060 PMC3338985

[B83] Izumi R. Yamada T. Yoshikai S. Sasaki H. Hattori M. Sakaki Y. (1992). Positive and negative regulatory elements for the expression of the Alzheimer’s disease amyloid precursor-encoding gene in mouse. *Gene* 112 189–195. 10.1016/0378-1119(92)90375-y 1555768

[B84] Jankowsky J. L. Fadale D. J. Anderson J. Xu G. M. Gonzales V. Jenkins N. A. (2004). Mutant presenilins specifically elevate the levels of the 42 residue beta-amyloid peptide *in vivo*: evidence for augmentation of a 42-specific gamma secretase. *Hum. Mol. Genet.* 13 159–170. 10.1093/hmg/ddh019 14645205

[B85] Janks L. Sharma C. V. R. Egan T. M. (2018). A central role for P2X7 receptors in human microglia. *J. Neuroinflammation* 15:325. 10.1186/s12974-018-1353-8 30463629 PMC6247771

[B86] Jiang C. Li G. Huang P. Liu Z. Zhao B. (2017). *The Gut Microbiota and Alzheimer’s Disease.* Amsterdam: IOS Press. 10.3233/JAD-161141 28372330

[B87] Jin H. Han J. Resing D. Liu H. Yue X. Miller R. L. (2018). Synthesis and in vitro characterization of a P2X7 radioligand [^123^I]TZ6019 and its response to neuroinflammation in a mouse model of Alzheimer disease. *Eur. J. Pharmacol.* 820 8–17. 10.1016/j.ejphar.2017.12.006 29225193 PMC5767129

[B88] Kim S. Y. Moon J. H. Lee H. G. Kim S. U. Lee Y. B. (2007). ATP released from beta-amyloid-stimulated microglia induces reactive oxygen species production in an autocrine fashion. *Exp. Mol. Med.* 39 820–827. 10.1038/emm.2007.89 18160853

[B89] Kindy M. S. Yu J. Guo J. T. Zhu H. (1999). Apolipoprotein serum amyloid A in Alzheimer’s disease. *J. Alzheimers Dis.* 1 155–167. 12214001 10.3233/jad-1999-1303

[B90] Koffie R. M. Meyer-Luehmann M. Hashimoto T. Adams K. W. Mielke M. L. Garcia-Alloza M. (2009). Oligomeric amyloid beta associates with postsynaptic densities and correlates with excitatory synapse loss near senile plaques. *Proc. Natl. Acad. Sci. U.S.A.* 106 4012–4017. 10.1073/pnas.0811698106 19228947 PMC2656196

[B91] Kondo T. Imamura K. Funayama M. Tsukita K. Miyake M. Ohta A. (2017). iPSC-based compound screening and in vitro trials identify a synergistic anti-amyloid β combination for Alzheimer’s disease. *Cell Rep.* 21 2304–2312. 10.1016/j.celrep.2017.10.109 29166618

[B92] Krasemann S. Madore C. Cialic R. Baufeld C. Calcagno N. El Fatimy R. (2017). The TREM2-APOE pathway drives the transcriptional phenotype of dysfunctional microglia in neurodegenerative diseases. *Immunity* 47 566–581.e9. 10.1016/j.immuni.2017.08.008 28930663 PMC5719893

[B93] Laliberte R. E. Eggle J. Gabel C. A. (1999). ATP treatment of human monocytes promotes caspase-1 maturation and externalization. *J. Biol. Chem.* 274 36944–36951. 10.1074/jbc.274.52.36944 10601248

[B94] Langfelder A. Okonji E. Deca D. Wei W. C. Glitsch M. D. (2015). Extracellular acidosis impairs P2Y receptor-mediated Ca^2+^ signalling and migration of microglia. *Cell Calcium* 57 247–256. 10.1016/j.ceca.2015.01.004 25623949 PMC6031298

[B95] Lansbury P. T. (1999). Evolution of amyloid: what normal protein folding may tell us about fibrillogenesis and disease. *Proc. Natl. Acad. Sci. U.S.A.* 96 3342–3344. 10.1073/pnas.96.7.3342 10097040 PMC34271

[B96] Lanz T. A. Carter D. B. Merchant K. M. (2003). Dendritic spine loss in the hippocampus of young PDAPP and Tg2576 mice and its prevention by the ApoE2 genotype. *Neurobiol. Dis.* 13 246–253. 10.1016/s0969-9961(03)00079-2 12901839

[B97] Larsen F. Gundersen G. Lopez R. Prydz H. (1992). CpG islands as gene markers in the human genome. *Genomics* 13 1095–1107. 10.1016/0888-7543(92)90024-m 1505946

[B98] Lee H. G. Won S. M. Gwag B. J. Lee Y. B. (2011). Microglial P2X(7) receptor expression is accompanied by neuronal damage in the cerebral cortex of the APPswe/PS1dE9 mouse model of Alzheimer’s disease. *Exp. Mol. Med.* 43 7–14. 10.3858/emm.2011.43.1.001 21088470 PMC3041940

[B99] Lee J. K. Jin H. K. Park M. H. Kim B. R. Lee P. H. Nakauchi H. (2014). Acid sphingomyelinase modulates the autophagic process by controlling lysosomal biogenesis in Alzheimer’s disease. *J. Exp. Med.* 211 1551–1570. 10.1084/jem.20132451 25049335 PMC4113944

[B100] Lee S. H. Le Pichon C. E. Adolfsson O. Gafner V. Pihlgren M. Lin H. (2016). Antibody-mediated targeting of Tau *in vivo* does not require effector function and microglial engagement. *Cell Rep.* 16 1690–1700. 10.1016/j.celrep.2016.06.099 27475227

[B101] Leon D. Sanchez-Nogueiro J. Marin-Garcia P. Miras-Portugal M. A. (2008). Glutamate release and synapsin-I phosphorylation induced by P2X7 receptors activation in cerebellar granule neurons. *Neurochem. Int.* 52 1148–1159. 10.1016/j.neuint.2007.12.004 18242779

[B102] Leon-Otegui M. Gomez-Villafuertes R. Diaz-Hernandez J. I. Diaz-Hernandez M. Miras-Portugal M. T. Gualix J. (2011). Opposite effects of P2X7 and P2Y2 nucleotide receptors on alpha-secretase-dependent APP processing in Neuro-2a cells. *FEBS Lett.* 585 2255–2262. 10.1016/j.febslet.2011.05.048 21651910

[B103] Ling Y. Morgan K. Kalsheker N. (2003). Amyloid precursor protein (APP) and the biology of proteolytic processing: relevance to Alzheimer’s disease. *Int. J. Biochem. Cell Biol.* 35 1505–1535. 10.1016/s1357-2725(03)00133-x 12824062

[B104] Liu G. David B. T. Trawczynski M. Fessler R. G. (2020). Advances in pluripotent stem cells: history, mechanisms, technologies, and applications. *Stem Cell Rev. Rep.* 16 3–32. 10.1007/s12015-019-09935-x 31760627 PMC6987053

[B105] Liu Q. Waltz S. Woodruff G. Ouyang J. Israel M. A. Herrera C. (2014). Effect of potent γ-secretase modulator in human neurons derived from multiple presenilin 1-induced pluripotent stem cell mutant carriers. *JAMA Neurol.* 71 1481–1489. 10.1001/jamaneurol.2014.2482 25285942 PMC4374637

[B106] Long J. M. Holtzman D. M. (2019). Alzheimer disease: an update on pathobiology and treatment strategies. *Cell* 179 312–339. 10.1016/j.cell.2019.09.001 31564456 PMC6778042

[B107] Lopez Lopez C. Tariot P. N. Caputo A. Langbaum J. B. Liu F. Riviere M. E. (2019). The Alzheimer’s prevention initiative generation program: study design of two randomized controlled trials for individuals at risk for clinical onset of Alzheimer’s disease. *Alzheimers Dement.* 5 216–227. 10.1016/j.trci.2019.02.005 31211217 PMC6562315

[B108] Lowe V. J. Lundt E. S. Albertson S. M. Przybelski S. A. Senjem M. L. Parisi J. E. (2019). Neuroimaging correlates with neuropathologic schemes in neurodegenerative disease. *Alzheimers Dement.* 15 927–939. 10.1016/j.jalz.2019.03.016 31175025 PMC6662599

[B109] Martin E. Amar M. Dalle C. Youssef I. Boucher C. Le Duigou C. (2019). New role of P2X7 receptor in an Alzheimer’s disease mouse model. *Mol. Psychiatry* 24 108–125. 10.1038/s41380-018-0108-3 29934546 PMC6756107

[B110] Martinez-Frailes C. Di Lauro C. Bianchi C. de Diego-Garcia L. Sebastian-Serrano A. Bosca L. (2019). Amyloid peptide induced neuroinflammation increases the P2X7 receptor expression in microglial cells, impacting on its functionality. *Front. Cell. Neurosci.* 13:143. 10.3389/fncel.2019.00143 31031598 PMC6474397

[B111] Matute C. Torre I. Perez-Cerda F. Perez-Samartin A. Alberdi E. Etxebarria E. (2007). P2X(7) receptor blockade prevents ATP excitotoxicity in oligodendrocytes and ameliorates experimental autoimmune encephalomyelitis. *J. Neurosci.* 27 9525–9533. 10.1523/JNEUROSCI.0579-07.2007 17728465 PMC6673129

[B112] McCarthy A. E. Yoshioka C. Mansoor S. E. (2019). Full-length P2X7 structures reveal how palmitoylation prevents channel desensitization. *Cell* 179 659–670.e13. 10.1016/j.cell.2019.09.017 31587896 PMC7053488

[B113] McGeer P. L. McGeer E. G. Yasojima K. (2000). Alzheimer disease and neuroinflammation. *J. Neural Transm. Suppl.* 59 53–57. 10961418 10.1007/978-3-7091-6781-6_8

[B114] McKhann G. M. Knopman D. S. Chertkow H. Hyman B. T. Jack CR Jr Kawas C. H. (2011). The diagnosis of dementia due to Alzheimer’s disease: recommendations from the National Institute on Aging-Alzheimer’s Association workgroups on diagnostic guidelines for Alzheimer’s disease. *Alzheimers Dement.* 7 263–269. 10.1016/j.jalz.2011.03.005 21514250 PMC3312024

[B115] McKinney C. E. (2017). Using induced pluripotent stem cells derived neurons to model brain diseases. *Neural Regen. Res.* 12 1062–1067. 28852383 10.4103/1673-5374.211180 PMC5558480

[B116] McLarnon J. G. Ryu J. K. Walker D. G. Choi H. B. (2006). Upregulated expression of purinergic P2X(7) receptor in Alzheimer disease and amyloid-beta peptide-treated microglia and in peptide-injected rat hippocampus. *J. Neuropathol. Exp. Neurol.* 65 1090–1097. 10.1097/01.jnen.0000240470.97295.d3 17086106

[B117] Meilandt W. J. Ngu H. Gogineni A. Lalehzadeh G. Lee S. H. Srinivasan K.. (2020). TREM2 deletion reduces late-stage amyloid plaque accumulation, elevates the Aβ42:Aβ40 ratio, and exacerbates axonal dystrophy and dendritic spine loss in the PS2APP Alzheimer’s mouse model. *J. Neurosci.* 40 1956–1974. 10.1523/JNEUROSCI.1871-19.2019 31980586 PMC7046459

[B118] Messemer N. Kunert C. Grohmann M. Sobottka H. Nieber K. Zimmermann H. (2013). P2X7 receptors at adult neural progenitor cells of the mouse subventricular zone. *Neuropharmacology* 73 122–137. 10.1016/j.neuropharm.2013.05.017 23727220

[B119] Miida T. Yamada T. Seino U. Ito M. Fueki Y. Takahashi A. (2006). Serum amyloid A (SAA)-induced remodeling of CSF-HDL. *Biochim. Biophys. Acta* 1761 424–433. 10.1016/j.bbalip.2006.03.013 16651021

[B120] Miras-Portugal M. T. Diaz-Hernandez M. Giraldez L. Hervas C. Gomez-Villafuertes R. Sen R. P. (2003). P2X7 receptors in rat brain: presence in synaptic terminals and granule cells. *Neurochem. Res.* 28 1597–1605. 10.1023/a:1025690913206 14570406

[B121] Miras-Portugal M. T. Sebastian-Serrano A. de Diego Garcia L. Diaz-Hernandez M. (2017). Neuronal P2X7 Receptor: involvement in neuronal physiology and pathology. *J. Neurosci.* 37 7063–7072. 10.1523/JNEUROSCI.3104-16.2017 28747389 PMC6705729

[B122] Morris J. C. Roe C. M. Grant E. A. Head D. Storandt M. Goate A. M. (2009). Pittsburgh compound B imaging and prediction of progression from cognitive normality to symptomatic Alzheimer disease. *Arch. Neurol.* 66 1469–1475. 10.1001/archneurol.2009.269 20008650 PMC2798814

[B123] Mucke L. Masliah E. Yu G. Q. Mallory M. Rockenstein E. M. Tatsuno G. (2000). High-level neuronal expression of abeta 1-42 in wild-type human amyloid protein precursor transgenic mice: synaptotoxicity without plaque formation. *J. Neurosci.* 20 4050–4058. 10.1523/jneurosci.20-11-04050.2000 10818140 PMC6772621

[B124] Mungenast A. E. Siegert S. Tsai L.-H. (2016). Modeling Alzheimer’s disease with human induced pluripotent stem (iPS) cells. *Mol. Cell. Neurosci.* 73 13–31. 10.1016/J.MCN.2015.11.010 26657644 PMC5930170

[B125] Munoz F. M. Gao R. Tian Y. Henstenburg B. A. Barrett J. E. Hu H. (2017). Neuronal P2X7 receptor-induced reactive oxygen species production contributes to nociceptive behavior in mice. *Sci. Rep.* 7:3539. 10.1038/s41598-017-03813-7 28615626 PMC5471238

[B126] NCBI (2017). *P2RX7 Purinergic Receptor P2X 7 [Homo sapiens (human)].* Available online at: https://www.ncbi.nlm.nih.gov/gene?Db=gene&Cmd=DetailsSearch&Term=5027 (accessed November 16, 2019).

[B127] Ni J. Wang P. Zhang J. Chen W. Gu L. (2013). Silencing of the P2X(7) receptor enhances amyloid-beta phagocytosis by microglia. *Biochem. Biophys. Res. Commun.* 434 363–369. 10.1016/j.bbrc.2013.03.079 23562658

[B128] Nicke A. Baumert H. G. Rettinger J. Eichele A. Lambrecht G. Mutschler E. (1998). P2X1 and P2X3 receptors form stable trimers: a novel structural motif of ligand-gated ion channels. *EMBO J.* 17 3016–3028. 10.1093/emboj/17.11.3016 9606184 PMC1170641

[B129] Niemi K. Teirila L. Lappalainen J. Rajamaki K. Baumann M. H. Oorni K. (2011). Serum amyloid A activates the NLRP3 inflammasome via P2X7 receptor and a cathepsin B-sensitive pathway. *J. Immunol.* 186 6119–6128. 10.4049/jimmunol.1002843 21508263

[B130] Novak P. Schmidt R. Kontsekova E. Zilka N. Kovacech B. Skrabana R. (2017). Safety and immunogenicity of the tau vaccine AADvac1 in patients with Alzheimer’s disease: a randomised, double-blind, placebo-controlled, phase 1 trial. *Lancet Neurol.* 16 123–134. 10.1016/S1474-4422(16)30331-3 27955995

[B131] Nuttle L. C. Dubyak G. R. (1994). Differential activation of cation channels and non-selective pores by macrophage P2z purinergic receptors expressed in Xenopus oocytes. *J. Biol. Chem.* 269 13988–13996. 7514597

[B132] O’Callaghan J. P. Sriram K. Miller D. B. (2008). Defining “neuroinflammation”. *Ann. N. Y. Acad. Sci.* 1139 318–330. 10.1196/annals.1432.032 18991877

[B133] Ochalek A. Mihalik B. Avci H. X. Chandrasekaran A. Téglási A. Bock I. (2017). Neurons derived from sporadic Alzheimer’s disease iPSCs reveal elevated TAU hyperphosphorylation, increased amyloid levels, and GSK3B activation. *Alzheimers Res. Ther.* 9:90. 10.1186/s13195-017-0317-z 29191219 PMC5709977

[B134] Ortiz-Virumbrales M. Moreno C. L. Kruglikov I. Marazuela P. Sproul A. Jacob S. (2017). CRISPR/Cas9-Correctable mutation-related molecular and physiological phenotypes in iPSC-derived Alzheimer’s PSEN2 N141I neurons. *Acta Neuropathol. Commun.* 5:77. 10.1186/s40478-017-0475-z 29078805 PMC5660456

[B135] Parvathenani L. K. Tertyshnikova S. Greco C. R. Roberts S. B. Robertson B. Posmantur R. (2003). P2X7 mediates superoxide production in primary microglia and is up-regulated in a transgenic mouse model of Alzheimer’s disease. *J. Biol. Chem.* 278 13309–13317. 10.1074/jbc.M209478200 12551918

[B136] Perez-Nievas B. G. Stein T. D. Tai H. C. Dols-Icardo O. Scotton T. C. Barroeta-Espar I. (2013). Dissecting phenotypic traits linked to human resilience to Alzheimer’s pathology. *Brain* 136(Pt 8), 2510–2526. 10.1093/brain/awt171 23824488 PMC3722351

[B137] Perl D. P. (2010). Neuropathology of Alzheimer’s disease. *Mt. Sinai J. Med.* 77 32–42. 10.1002/msj.20157 20101720 PMC2918894

[B138] Perregaux D. G. McNiff P. Laliberte R. Conklyn M. Gabel C. A. (2000). ATP acts as an agonist to promote stimulus-induced secretion of IL-1 beta and IL-18 in human blood. *J. Immunol.* 165 4615–4623. 10.4049/jimmunol.165.8.4615 11035104

[B139] Petrilli V. Papin S. Dostert C. Mayor A. Martinon F. Tschopp J. (2007). Activation of the NALP3 inflammasome is triggered by low intracellular potassium concentration. *Cell Death Differ.* 14 1583–1589. 10.1038/sj.cdd.4402195 17599094

[B140] Price D. L. Sisodia S. S. (1998). Mutant genes in familial Alzheimer’s disease and transgenic models. *Annu. Rev. Neurosci.* 21 479–505. 10.1146/annurev.neuro.21.1.479 9530504

[B141] Qin J. Zhang X. Wang Z. Li J. Zhang Z. Gao L. (2017). Presenilin 2 deficiency facilitates Abeta-induced neuroinflammation and injury by upregulating P2X7 expression. *Sci. China Life Sci.* 60 189–201. 10.1007/s11427-016-0347-4 28120269

[B142] Ransohoff R. M. (2016). A polarizing question: do M1 and M2 microglia exist? *Nat. Neurosci.* 19 987–991. 10.1038/nn.4338 27459405

[B143] Rassendren F. Buell G. N. Virginio C. Collo G. North R. A. Surprenant A. (1997). The permeabilizing ATP receptor, P2X7. Cloning and expression of a human cDNA. *J. Biol. Chem.* 272 5482–5486. 10.1074/jbc.272.9.5482 9038151

[B144] Ribeiro D. E. Roncalho A. L. Glaser T. Ulrich H. Wegener G. Joca S. (2019). P2X7 receptor signaling in stress and depression. *Int. J. Mol. Sci.* 20:E2778. 10.3390/ijms20112778 31174279 PMC6600521

[B145] Rigato C. Swinnen N. Buckinx R. Couillin I. Mangin J. M. Rigo J. M. (2012). Microglia proliferation is controlled by P2X7 receptors in a Pannexin-1-independent manner during early embryonic spinal cord invasion. *J. Neurosci.* 32 11559–11573. 10.1523/jneurosci.1042-12.2012 22915101 PMC6703767

[B146] Ryu J. K. McLarnon J. G. (2008). Block of purinergic P2X(7) receptor is neuroprotective in an animal model of Alzheimer’s disease. *Neuroreport* 19 1715–1719. 10.1097/WNR.0b013e3283179333 18852683

[B147] Saez-Orellana F. Fuentes-Fuentes M. C. Godoy P. A. Silva-Grecchi T. Panes J. D. Guzman L. (2018). P2X receptor overexpression induced by soluble oligomers of amyloid beta peptide potentiates synaptic failure and neuronal dyshomeostasis in cellular models of Alzheimer’s disease. *Neuropharmacology* 128 366–378. 10.1016/j.neuropharm.2017.10.027 29079292 PMC5858180

[B148] Saez-Orellana F. Godoy P. A. Bastidas C. Y. Silva-Grecchi T. Guzman L. Aguayo L. G. (2016). ATP leakage induces P2XR activation and contributes to acute synaptic excitotoxicity induced by soluble oligomers of beta-amyloid peptide in hippocampal neurons. *Neuropharmacology* 100 116–123. 10.1016/j.neuropharm.2015.04.005 25896766

[B149] Salloway S. Sperling R. Fox N. C. Blennow K. Klunk W. Raskind M. (2014). Two phase 3 trials of bapineuzumab in mild-to-moderate Alzheimer’s disease. *N. Engl. J. Med.* 370 322–333. 10.1056/NEJMoa1304839 24450891 PMC4159618

[B150] Samways D. S. Li Z. Egan T. M. (2014). Principles and properties of ion flow in P2X receptors. *Front Cell. Neurosci.* 8:6. 10.3389/fncel.2014.00006 24550775 PMC3914235

[B151] Sanz J. M. Chiozzi P. Ferrari D. Colaianna M. Idzko M. Falzoni S. (2009). Activation of microglia by amyloid {beta} requires P2X7 receptor expression. *J. Immunol.* 182 4378–4385. 10.4049/jimmunol.0803612 19299738

[B152] Sanz J. M. Falzoni S. Rizzo R. Cipollone F. Zuliani G. Di Virgilio F. (2014). Possible protective role of the 489C>T P2X7R polymorphism in Alzheimer’s disease. *Exp. Gerontol.* 60 117–119. 10.1016/j.exger.2014.10.009 25456845 PMC4266448

[B153] Sebastian-Serrano A. de Diego-Garcia L. di Lauro C. Bianchi C. Diaz-Hernandez M. (2019). Nucleotides regulate the common molecular mechanisms that underlie neurodegenerative diseases; therapeutic implications. *Brain Res. Bull.* 151 84–91. 10.1016/j.brainresbull.2019.01.031 30721769

[B154] Selkoe D. J. (2001). Alzheimer’s disease: genes, proteins, and therapy. *Physiol. Rev.* 81 741–766. 10.1152/physrev.2001.81.2.741 11274343

[B155] Selkoe D. J. (2002). Alzheimer’s disease is a synaptic failure. *Science* 298 789–791. 10.1126/science.1074069 12399581

[B156] Selkoe D. J. (2019). Alzheimer disease and aducanumab: adjusting our approach. *Nat. Rev. Neurol.* 15 365–366. 10.1038/s41582-019-0205-1 31138932

[B157] Shankar G. M. Li S. Mehta T. H. Garcia-Munoz A. Shepardson N. E. Smith I. (2008). Amyloid-beta protein dimers isolated directly from Alzheimer’s brains impair synaptic plasticity and memory. *Nat. Med.* 14 837–842. 10.1038/nm1782 18568035 PMC2772133

[B158] Siegfried Z. Eden S. Mendelsohn M. Feng X. Tsuberi B. Z. Cedar H. (1999). DNA methylation represses transcription *in vivo*. *Nat. Genet.* 22 203–206. 10.1038/9727 10369268

[B159] Skarratt K. K. Fuller S. J. Sluyter R. Dao-Ung L. P. Gu B. J. Wiley J. S. (2005). A 5’ intronic splice site polymorphism leads to a null allele of the P2X7 gene in 1-2% of the Caucasian population. *FEBS Lett.* 579 2675–2678. 10.1016/j.febslet.2005.03.091 15862308

[B160] Skarratt K. K. Gu B. J. Lovelace M. D. (2020). A P2RX7 single nucleotide polymorphism haplotype promotes exon 7 and 8 skipping and disrupts receptor function. *FASEB J.* 34 3884–3901. 10.1096/fj.201901198RR 32003498

[B161] Sluyter R. Stokes L. (2011). Significance of P2X7 receptor variants to human health and disease. *Recent Pat. DNA Gene Seq.* 5 41–54. 10.2174/187221511794839219 21303345

[B162] Song W. M. Colonna M. (2018). The identity and function of microglia in neurodegeneration. *Nat. Immunol.* 19 1048–1058. 10.1038/s41590-018-0212-1 30250185

[B163] Sperlagh B. Kofalvi A. Deuchars J. Atkinson L. Milligan C. J. Buckley N. J. (2002). Involvement of P2X7 receptors in the regulation of neurotransmitter release in the rat hippocampus. *J. Neurochem.* 81 1196–1211. 10.1046/j.1471-4159.2002.00920.x 12068068

[B164] Stockley J. H. O’Neill C. (2008). Understanding BACE1: essential protease for amyloid-beta production in Alzheimer’s disease. *Cell. Mol. Life Sci.* 65 3265–3289. 10.1007/s00018-008-8271-3 18695942 PMC11131673

[B165] Sullivan S. E. Young-Pearse T. L. (2017). Induced pluripotent stem cells as a discovery tool for Alzheimer×s disease. *Brain Res.* 1656 98–106. 10.1016/J.BRAINRES.2015.10.005 26459988 PMC4833689

[B166] Surprenant A. Rassendren F. Kawashima E. North R. A. Buell G. (1996). The cytolytic P2Z receptor for extracellular ATP identified as a P2X receptor (P2X7). *Science* 272 735–738. 10.1126/science.272.5262.735 8614837

[B167] Takahashi K. Tanabe K. Ohnuki M. Narita M. Ichisaka T. Tomoda K. (2007). Induction of pluripotent stem cells from adult human fibroblasts by defined factors. *Cell* 131 861–872. 10.1016/j.cell.2007.11.019 18035408

[B168] Takeda K. Akira S. (2004). TLR signaling pathways. *Semin. Immunol.* 16 3–9. 10.1016/j.smim.2003.10.003 14751757

[B169] Thawkar B. S. Kaur G. (2019). Inhibitors of NF-kappaB and P2X7/NLRP3/Caspase 1 pathway in microglia: Novel therapeutic opportunities in neuroinflammation induced early-stage Alzheimer’s disease. *J. Neuroimmunol.* 326 62–74. 10.1016/j.jneuroim.2018.11.010 30502599

[B170] Theunis C. Crespo-Biel N. Gafner V. Pihlgren M. López-Deber M. P. Reis P. (2013). Efficacy and safety of a liposome-based vaccine against protein Tau, assessed in Tau.P301L mice that model tauopathy. *PLoS One* 8:e72301. 10.1371/journal.pone.0072301 23977276 PMC3747157

[B171] Tsao H. K. Chiu P. H. Sun S. H. (2013). PKC-dependent ERK phosphorylation is essential for P2X7 receptor-mediated neuronal differentiation of neural progenitor cells. *Cell Death Dis.* 4:e751. 10.1038/cddis.2013.274 23907465 PMC3763436

[B172] Tyler S. J. Dawbarn D. Wilcock G. K. Allen S. J. (2002). alpha- and beta-secretase: profound changes in Alzheimer’s disease. *Biochem. Biophys. Res. Commun.* 299 373–376. 10.1016/s0006-291x(02)02635-9 12445809

[B173] Um J. W. Kaufman A. C. Kostylev M. Heiss J. K. Stagi M. Takahashi H. (2013). Metabotropic glutamate receptor 5 is a coreceptor for Alzheimer abeta oligomer bound to cellular prion protein. *Neuron* 79 887–902. 10.1016/j.neuron.2013.06.036 24012003 PMC3768018

[B174] Usenovic M. Niroomand S. Drolet R. E. Yao L. Gaspar R. C. Hatcher N. G. (2015). Internalized tau oligomers cause neurodegeneration by inducing accumulation of pathogenic tau in human neurons derived from induced pluripotent stem cells. *J. Neurosci.* 35 14234–14250. 10.1523/JNEUROSCI.1523-15.2015 26490863 PMC6605424

[B175] Venigalla M. Sonego S. Gyengesi E. Sharman M. J. Munch G. (2016). Novel promising therapeutics against chronic neuroinflammation and neurodegeneration in Alzheimer’s disease. *Neurochem. Int.* 95 63–74. 10.1016/j.neuint.2015.10.011 26529297

[B176] Vermunt L. Sikkes S. A. M. van den Hout A. Handels R. Bos I. van der Flier W. M. (2019). Duration of preclinical, prodromal, and dementia stages of Alzheimer’s disease in relation to age, sex, and APOE genotype. *Alzheimers Dement.* 15 888–898. 10.1016/j.jalz.2019.04.001 31164314 PMC6646097

[B177] Virginio C. MacKenzie A. Rassendren F. A. North R. A. Surprenant A. (1999). Pore dilation of neuronal P2X receptor channels. *Nat. Neurosci.* 2 315–321. 10.1038/7225 10204537

[B178] Vogels T. Murgoci A.-N. Hromádka T. (2019). Intersection of pathological tau and microglia at the synapse. *Acta Neuropathol. Commun.* 7:109. 10.1186/s40478-019-0754-y 31277708 PMC6612163

[B179] Vogt N. M. Kerby R. L. Dill-McFarland K. A. Harding S. J. Merluzzi A. P. Johnson S. C. (2017). Gut microbiome alterations in Alzheimer’s disease. *Sci. Rep.* 7:13537. 10.1038/s41598-017-13601-y 29051531 PMC5648830

[B180] Walker D. G. Lue L. F. (2015). Immune phenotypes of microglia in human neurodegenerative disease: challenges to detecting microglial polarization in human brains. *Alzheimers Res. Ther.* 7:56. 10.1186/s13195-015-0139-9 26286145 PMC4543480

[B181] Wang H. Y. Lee D. H. D’Andrea M. R. Peterson P. A. Shank R. P. Reitz A. B. (2000). beta-Amyloid(1-42) binds to alpha7 nicotinic acetylcholine receptor with high affinity. Implications for Alzheimer’s disease pathology. *J. Biol. Chem.* 275 5626–5632. 10.1074/jbc.275.8.5626 10681545

[B182] Wang S. Jing H. Yang H. Liu Z. Guo H. Chai L. (2015). Tanshinone I selectively suppresses pro-inflammatory genes expression in activated microglia and prevents nigrostriatal dopaminergic neurodegeneration in a mouse model of Parkinson’s disease. *J. Ethnopharmacol.* 164 247–255. 10.1016/j.jep.2015.01.042 25666429

[B183] Wang W. Y. Tan M. S. Yu J. T. Tan L. (2015). Role of pro-inflammatory cytokines released from microglia in Alzheimer’s disease. *Ann. Transl. Med.* 3:136. 10.3978/j.issn.2305-5839.2015.03.49 26207229 PMC4486922

[B184] Wang Y. Cella M. Mallinson K. Ulrich J. D. Young K. L. Robinette M. L. (2015). TREM2 lipid sensing sustains the microglial response in an Alzheimer’s disease model. *Cell* 160 1061–1071. 10.1016/j.cell.2015.01.049 25728668 PMC4477963

[B185] Wischik C. M. Edwards P. C. Lai R. Y. K. Roth M. Harrington C. R. (1996). Selective inhibition of Alzheimer disease-like tau aggregation by phenothiazines. *Proc. Natl. Acad. Sci. U.S.A.* 93 11213–11218. 10.1073/pnas.93.20.11213 8855335 PMC38310

[B186] Wischik C. M. Staff R. T. Wischik D. J. Bentham P. Murray A. D. Storey J. M. D. (2015). Tau aggregation inhibitor therapy: an exploratory phase 2 study in mild or moderate Alzheimer’s disease. *J. Alzheimers Dis.* 44 705–720. 10.3233/JAD-142874 25550228

[B187] Yanamandra K. Jiang H. Mahan T. E. Maloney S. E. Wozniak D. F. Diamond M. I. (2015). Anti-tau antibody reduces insoluble tau and decreases brain atrophy. *Ann. Clin. Transl. Neurol.* 2 278–288. 10.1002/acn3.176 25815354 PMC4369277

[B188] Ye L. Muench M. O. Fusaki N. Beyer A. I. Wang J. Qi Z. (2013). Blood cell-derived induced pluripotent stem cells free of reprogramming factors generated by Sendai viral vectors. *Stem Cells Transl. Med.* 2 558–566. 10.5966/sctm.2013-0006 23847002 PMC3726135

[B189] Yoshiyama Y. Higuchi M. Zhang B. Huang S. M. Iwata N. Saido T. C. (2007). Synapse loss and microglial activation precede tangles in a P301S tauopathy mouse model. *Neuron* 53 337–351. 10.1016/j.neuron.2007.01.010 17270732

[B190] Yuskaitis C. J. Jope R. S. (2009). Glycogen synthase kinase-3 regulates microglial migration, inflammation, and inflammation-induced neurotoxicity. *Cell. Signal.* 21 264–273. 10.1016/j.cellsig.2008.10.014 19007880 PMC2630396

[B191] Zhang C. Rissman R. A. Feng J. (2015). Characterization of ATP alternations in an Alzheimer’s disease transgenic mouse model. *J. Alzheimers. Dis.* 44 375–378. 10.3233/JAD-141890 25261448 PMC4305018

[B192] Zhang D. Pekkanen-Mattila M. Shahsavani M. Falk A. Teixeira A. I. Herland A. (2014). A 3D Alzheimer’s disease culture model and the induction of P21-activated kinase mediated sensing in iPSC derived neurons. *Biomaterials* 35 1420–1428. 10.1016/j.biomaterials.2013.11.028 24290439

[B193] Zuo L. Zhou T. Pannell B. K. Ziegler A. C. Best T. M. (2015). Biological and physiological role of reactive oxygen species–the good, the bad and the ugly. *Acta Physiol.* 214 329–348. 10.1111/apha.12515 25912260

